# Active and tunable nanophotonic metamaterials

**DOI:** 10.1515/nanoph-2022-0188

**Published:** 2022-08-17

**Authors:** Kebin Fan, Richard D. Averitt, Willie J. Padilla

**Affiliations:** School of Electronic Science and Engineering, Nanjing University, Nanjing 210023, China; Department of Physics, UC San Diego, La Jolla, CA 92093, USA; Department of Electrical and Computer Engineering, Duke University, Durham, NC 27708, USA

**Keywords:** dynamic, electromagnetic, metamaterials, metasurfaces, nano, photonic, tunable

## Abstract

Metamaterials enable subwavelength tailoring of light–matter interactions, driving fundamental discoveries which fuel novel applications in areas ranging from compressed sensing to quantum engineering. Importantly, the metallic and dielectric resonators from which static metamaterials are comprised present an open architecture amenable to materials integration. Thus, incorporating responsive materials such as semiconductors, liquid crystals, phase-change materials, or quantum materials (e.g., superconductors, 2D materials, etc.) imbue metamaterials with dynamic properties, facilitating the development of active and tunable devices harboring enhanced or even entirely novel electromagnetic functionality. Ultimately, active control derives from the ability to craft the local electromagnetic fields; accomplished using a host of external stimuli to modify the electronic or optical properties of the responsive materials embedded into the active regions of the subwavelength resonators. We provide a broad overview of this frontier area of metamaterials research, introducing fundamental concepts and presenting control strategies that include electronic, optical, mechanical, thermal, and magnetic stimuli. The examples presented range from microwave to visible wavelengths, utilizing a wide range of materials to realize spatial light modulators, effective nonlinear media, on-demand optics, and polarimetric imaging as but a few examples. Often, active and tunable nanophotonic metamaterials yield an emergent electromagnetic response that is more than the sum of the parts, providing reconfigurable or real-time control of the amplitude, phase, wavevector, polarization, and frequency of light. The examples to date are impressive, setting the stage for future advances that are likely to impact holography, beyond 5G communications, imaging, and quantum sensing and transduction.

## 1 Motivation

Photonics is the science and technology of light and impacts myriad areas of modern society including health, lighting and energy, communications, safety and security, and manufacturing [1, 2]. Moreover, the importance and necessity for photonics innovation continues to grow as society becomes more digitally connected yet environmentally aware. Equally fertile is the field of nanotechnology which involves the manipulation of matter at near-atomic scales to realize new materials and devices and has also made a broad impact in the societal areas mentioned above [3]. By extension, nanophotonics is the investigation of novel light–matter interactions to generate, guide, amplify, and detect light at the nanoscale [4, 5]. As a field, nanophotonics has flourished and spans physics, chemistry, engineering, materials science, bioscience and, increasingly, computer and data science. Operationally, nanophotonics may be organized into four broad areas: optical and physical phenomena, materials, integration, and devices and systems. Indeed, nanophotonics is vast and includes a diversity of topics such as plasmonics and photonic bandgap materials utilizing a host of materials encompassing metals, insulators, semiconductor heterostructures, and two-dimensional van der Waals (2D-vdW) materials [6–8]. Often, nanophotonics is thought of as spanning the visible spectral range of wavelengths but is arguably more expansive, extending from microwave through X-rays wavelengths when considering the prevalence of nanoengineered materials utilized over this spectral range. As an example, semiconductor heterostructures and, more recently, graphene and other 2D-vdW, have been used to create and demonstrate as host of photon-based technologies spanning this range [9–12].

Metamaterials have emerged as an archetype of spectrum-spanning photonic and nanophotonic materials where rational design principles based on Maxwell’s equations are coupled with state-of-the-art computation, fabrication, and characterization strategies [13, 14]. Specifically, metamaterials enable subwavelength tailoring of light–matter interactions and can be classified as artificially structured materials that exhibit extraordinary electromagnetic properties not available or not easily obtainable in nature. This is accomplished through sub-wavelength patterning of unit cells to realize a desired electromagnetic response encoded in the effective permittivity and permeability. The experimental verification of a negative refractive index generated significant interest in electromagnetic metamaterials, captivating the interest of many scientists, engineers, and materials scientists [15]. Numerous applications have emerged over the last two decades, ranging from satellite communications to imaging [16–18]. The proliferation of metamaterials concepts and devices has continued at an impressive pace with a heavy emphasis on two-dimensional metamaterials, commonly referred to as metasurfaces [19–21]. In the following, we narrow the focus of discussion to metamaterials (this term is used throughout the review to include metasurfaces) that are dynamic which provides an immense level of functionality to advance the aforementioned societal goals of nanophotonics.

The subwavelength unit cells that comprise metamaterials (typically either metallic or dielectric resonators are described in more detail in Section 2) present an open architecture amenable to materials integration. Thus, the incorporation of functional materials including semiconductors, liquid crystals, phase-change materials, or quantum materials enable dynamic metamaterials, aiding demonstration of novel devices with extraordinary electromagnetic properties. Active metamaterial control derives from the ability to craft the local electromagnetic fields; accomplished using external stimulus to modify the electronic or optical properties of the responsive materials embedded into the active regions of the subwavelength resonators. The goal of this review is to provide specific examples and details of active metamaterials that have been realized and the routes by which various stimuli can exert control.

**Figure 1:**
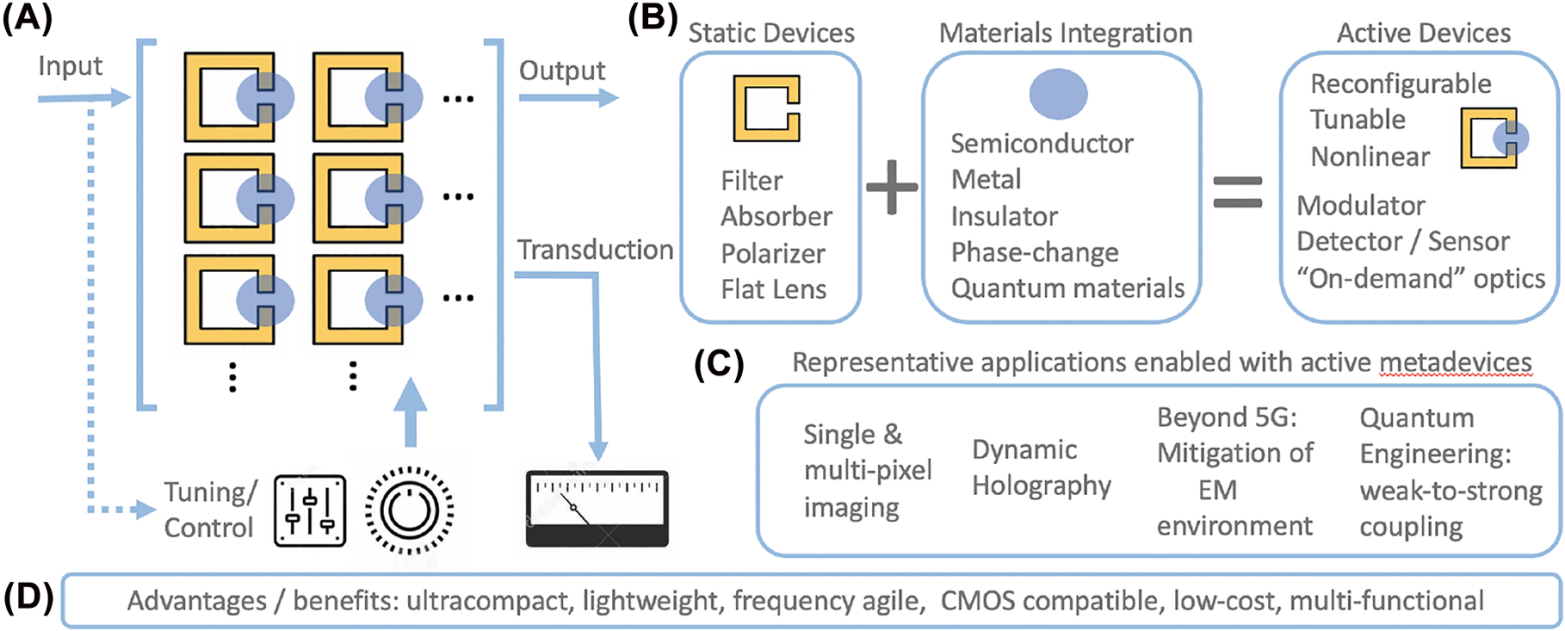
Schematic overview of active metamaterials. (A) Incident electromagnetic radiation (the input) interacts with a metamaterial structure to modify the output characteristics of the radiation. Material integration (blue circles) allows for tuning of the metamaterial response to control the output or enable signal transduction. The input could also modify the metamaterial response (e.g., a nonlinear interaction). (B) Materials integration imbues static metamaterials with electromagnetic properties that can be tuned or controlled. (C) Examples of potential applications of active metamaterials and (D) benefits of active metamaterials design.

However, before launching into these details, a conceptualization of active metamaterials is presented in Figure 1. Figure 1(A) shows an array of subwavelength resonators (in this case, depicted as gold “C-chape” resonators which could be, in principle, dielectric resonators or any other type of subwavelength structure). The blue dots represent a responsive material that has been integrated with the metamaterial to enable control through modification of the local dielectric environment. As shown in Figure 1B, an otherwise static (but still functional) metamaterial can be accorded active, dynamic, or reconfigurable functionality with targeted materials integration. As examples, a filter could serve as a modulator, the retardation of a metamaterial polarizer could be tuned, or the focus of a metasurface lens could be shifted. The versatility of this platform is enormous and is determined by the choice of material (Figure 1(B)) and the method of stimulus which includes electrical, optical, thermal, mechanical, or magnetic routes (or, in some cases, a combination thereof). The characteristics of the input (amplitude, direction, polarization, frequency) are actively modified upon interacting with the active metamaterial to achieve the desired output. Alternatively, instead of an augmented propagating field, transduction could be accomplished where the input is converted to a current, voltage, temperature, etc., enabling, for example, enhanced sensing or detection. Finally, we note that the input field could actuate the active metamaterial (indicated by dashed line in Figure 1(A)) through a nonlinear interaction, resulting in a modified output or transduced signal. A plethora of applications (some realized, some future aspirational goals) are presented in Figure 1(C) and include single or multi-pixel imaging and active control of the electromagnetic environment, of relevance to the reliability of future communication systems.

The power of metamaterials to create active electromagnetic devices stems from a rational design approach that is relatively wavelength agnostic. Moreover, concrete advantages (Figure 1(D)) include compact and lightweight “on-demand” optics, and integrability with existing technologies (e.g., CMOS). As nanophotonic metamaterials continue to evolve more emphasis will naturally be placed on achieving realistic applications. Although nanophotonic metamaterials have demonstrated novel electromagnetic properties, further enhancing them with dynamic properties would certainly bolster this goal. Before we discuss the scope and content of this review, we note that some common terms used to describe the real-time changeable properties of nanophotonic metamaterials include: *dynamic*, *tunable*, and *active*, and we use these terms throughout.

In this brief review it is not possible to cover all of the important results in the area of active metamaterials. The field of nanophotonic metamaterials is still fast growing and, although compiling an exhaustive list of representative works is a futile effort, we refer interested readers to the following review papers on active, dynamic, and tunable metasurfaces [22–30]. Our intent is to provide examples that highlight the underlying principles with a broad view of what has been accomplished to help set the stage for future research directions. Section 2 is a brief introduction to subwavelength metallic and dielectric resonators from which static metamaterials are created. This provides for understanding how materials integration enables control. Section 3 gives an overview of the stimulus routes that are available to control the electromagnetic properties of metamaterials. This section also presents examples of responsive materials that have been integrated with metamaterial architectures. This is followed by a discussion of devices and applications in Section 4 which also includes a summary and possible future directions of broad interest.

## 2 Artificial photonic materials

In this section, a brief overview of static artificial photonic electromagnetic materials (APM) is presented. The primary objective is to describe the basic properties of metallic and dielectric materials en route to active metamaterials. Nonetheless, a broader perspective (hence the term APM) is presented since APMs beyond metallic and dielectric metamaterials are amenable to dynamic control strategies through materials integration. It just so happens that the majority of active metamaterials that have been investigated are metallic and dielectric metasurfaces.

Structured material systems have long been used to tailor electromagnetic responses and have enabled the manipulation of electromagnetic waves beyond what is possible with natural materials. Generally, configurations of metallic, dielectric, or metallodielectric materials in one, two, and/or three dimensions are termed artificial photonic electromagnetic materials (APMs). The field of APMs is broad, and common sub-classes include: metallic metamaterials, all-dielectric metamaterials, photonic crystals, plasmonics, and artificial dielectrics. Many APMs obey the principle of *electromagnetic similitude* [31, 32], where geometric scaling enables operation across a broad swath of the electromagnetic spectrum. APMs with characteristic dimensions of few hundred nanometers or smaller (1–100 nm), can be classified as nanophotonic materials. APMs can be differentiated through comparison of their characteristic geometrical feature size (*p*) to the wavelength of free-space light at which they are resonant (*λ*_0_). For example, for metallic and all-dielectric metamaterials and plasmonics *p* is the side length or diameter, whereas for photonic crystals and artificial dielectrics *p* is the periodicity. We take the dimensionless parameter *R* to be the free-space wavelength to characteristic feature size, i.e. *R* = *λ*_0_/*p* to distinguish between different APMs [33].

Metal-based metamaterials are typically in the range of *R* ≳ 10, whereas all-dielectric metasurfaces achieve *R* ≈ 2 − 5 [13, 34]. Localized surface plasmon structures in the infrared and optical regime are in the same operational range as metamaterials, i.e. *R* ≳ 10 [6, 35]. Photonic crystals – also termed electromagnetic band gap materials – usually operate in the range 1 ≲ *R* ≲ 2 [7, 36]. Early demonstrations of artificial dielectric lenses consisted of disconnected metallic plates which were separated by several wavelengths in the electric field direction i.e. *R* ≳ 0.07, but with small spacing in the magnetic field direction *R* ≈ 10 [37, 38]. Alternative artificial dielectric lens designs used *R* ≳ 10 in all directions. We note that the different types of APMs operate in different ranges of wavelength to characteristic feature size [20], often designated as: dispersive effective media (2 < *R* < 100), and Floquet–Bloch (*R* ≤ 2) [39]. The term *effective media* (*R* ≥ 100) [40], is usually reserved for composites of materials that can be described by classical mixing formulas [41], although electromagnetic metamaterials have achieved *R* > 10^3^ [42, 43].

In the following section, specific operational characteristics of metallic and dielectric metamaterials are described with an emphasis on providing an understanding of how, as described in subsequent sections, materials integration facilitates active control.

### 2.1 Electromagnetic metamaterials and metasurfaces

**Figure 2:**
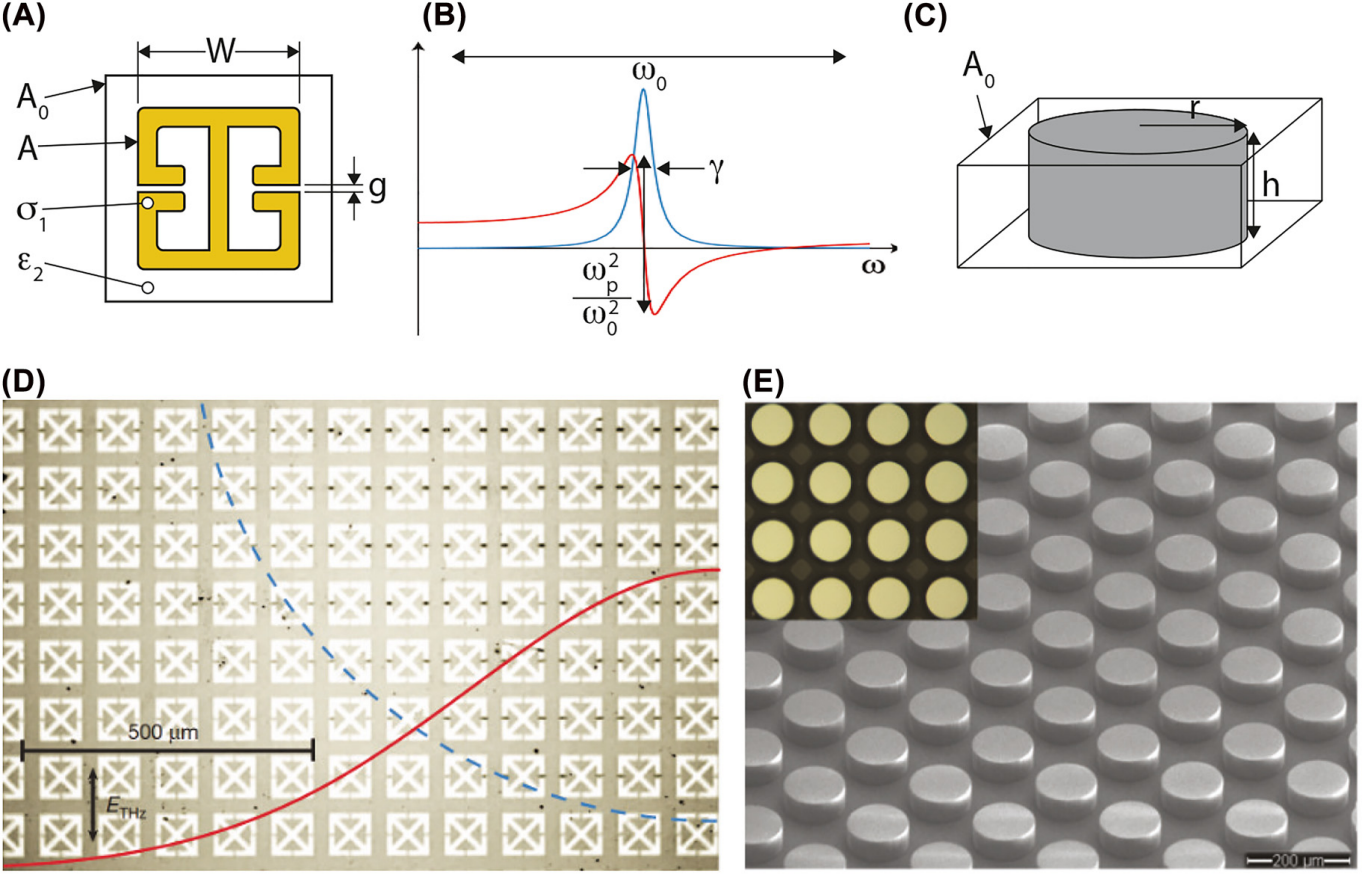
Geometry of metallic and dielectric unit cells that determine the resonant frequency and corresponding electromagnetic response of metamaterials. (A) and (C) Show a unit-cell of a metallic metamaterial and all-dielectric metasurface, respectively, with key geometric parameters specified. (B) A frequency dependent resonant material response in the permittivity and/or permeability is obtained from a macroscopic material fashioned from the unit-cells shown in (A) and (C). Images of a metallic metamaterial (D) and all-dielectric metasurface (E) are shown both of which are resonant in the THz region of the spectrum. In (D) the SRR array is deposited on top of VO_2_ which is a phase change material exhibiting an insulator-to-metal transition at ∼340 K. The blue dashed line shows a quarter of the THz beam which has a Gaussian profile as indicated by the red line. The black regions in the capacitive gaps arise from electric field enhancement that is sufficient to drive the transition where the subsequent Joule heating damages the VO_2_. Close inspection reveals that the damage tracks the Gaussian beam profile. Reprinted from [54], Copyright 2012, Nature Publishing Group. (E) Reprinted from [55], Copyright 2017, Optica.

Electromagnetic metamaterials and metasurfaces consist of arrays of structured sub-wavelength metallic or dielectric elements, (see Figure 2) and are often described as possessing a frequency-dependent effective permittivity *ϵ*_eff_(*ω*), and/or effective permeability μ_eff_(*ω*) [13, 34, 44]. Initial interest in metamaterials arose due to their ability to exhibit exotic electromagnetic effects, including negative refractive index [15], and other novel properties, some of which are difficult to achieve with natural materials. Initial demonstrations of electromagnetic metamaterials were carried out at microwave frequencies [45], but were quickly scaled to lower [46] and higher ranges, including millimeter wave, terahertz [47], mid infrared [48], near infrared [49], and visible [50]. The scaling of metamaterials throughout the spectrum highlights the principle of electromagnetic similitude described above [31].

#### 2.1.1 Metallic metamaterials

Metamaterial components are typically fashioned from highly conductive metals, such as copper, gold, silver, or aluminum, and formed into periodic arrays of wires [51] or various types of split ring resonators (see Figure 2A for an example) [52]. A unit-cell may consist of a single split ring resonator (SRR), or may consist of several identical or dissimilar sub-units, which are then arrayed to fill space in one, two, or three dimensions (Figure 2D shows an image of an SRR metasurface with additional details of the particular image described later in the review). A key feature of metallic-based metamaterials is that the subwavelength structure exhibits a resonant response that arises from the geometrically defined capacitance and inductance. That is, an SRR is essentially an LC circuit and is well-described as a Lorentz oscillator (or Lorentzian) [53]. A salient feature – termed the metamaterial design paradigm – is that the geometry of the unit-cells governs the Lorentz oscillator parameters for both *ϵ*_eff_(*ω*), and μ_eff_(*ω*), and thus the electromagnetic scattering properties. For example, the permittivity is given as

(1) $$\epsilon_{\text{eff}}\left(\omega \right)=\epsilon_{\infty }+\frac{\omega_{\text{p}}^{2}}{\omega_{0}^{2}-\omega^{2}-i\omega \gamma }$$

where *ϵ*_∞_ gives contributions to the permittivity outside of the frequency range of consideration, $\omega_{\text{p}}^{2}$ is the plasma frequency squared, *ω*_0_ is the resonance frequency, and *γ* is the scattering frequency. The metamaterial resonance frequency, *ω*_0_ ≈ (*LC*)^−1/2^, is governed by the capacitance (*C*), and inductance (*L*) given by the gap (Figure 2A – with the dimension labeled as *g*) and wire self inductance of the unit-cell, respectively. The total length *ℓ* of the metallic unit-cell element determines the resonance wavelength *λ*_0_, and thus the resonance frequency, i.e. $\omega_{0}=2\pi c\lambda_{0}^{-1}$ , where *c* is the speed of light in vacuum. In particular, a metallic metamaterial unit-cell is equivalent to a half-wave dipole antenna, where *ℓ* = *λ*_0_/2. The oscillator strength defined as $\omega_{\text{p}}^{2}/\omega_{0}^{2}$ , (see Eq. (1)) is proportional to the metal areal density (*A*/*A*_0_ where *A*_0_ is the unit-cell area and *A* is the SRR area), and determines peak and minimum values of *ϵ*_eff_(*ω*) near *ω*_0_. The resonance width *γ* is governed by the intrinsic loss of the materials (e.g., the real part of the conductivity *σ*_1_ of the metal comprising the SRR and the loss of the local dielectric environment given as *ϵ*_2_ – see Figure 2(A)) used to fashion the resonator. Figure 2(B) plots a representative Lorentzian *ϵ*_eff_(*ω*) = *ϵ*_eff,1_(*ω*) + *iϵ*_eff,2_(*ω*). The red line is *ϵ*_eff,1_(*ω*) and the blue line is *ϵ*_eff,2_(*ω*). A similar Lorentzian response describes the effective permeability μ_eff_(*ω*). It is important to note that the resonant response can depend on the orientation of the incident electromagnetic field with respect to the SRR (i.e. the effective *ϵ*_eff_(*ω*) and μ_eff_(*ω*) are tensors with components determined by the appropriate point group for a given structure) [56].

For an SRR on resonance, the electric field is reasonably well-confined within the split-ring capacitive gap which has dimensions 20–50 times smaller than the SRR. For example, for an SRR that is resonant in the terahertz region (e.g., ∼1 THz) with a square unit cell (refer to Figure 2A), a typical periodicity $A_{0}^{1/2}$ is 50 μm, with an SRR sidelength *W* ∼ 40 μm and capacitive gap *g* ∼ 2 μm. Moreover, because of the localized electric field there is, to leading order, very little interaction with neighboring unit-cells. Thus, the resonant response depicted in Figure 2(B) is largely determined by the geometry of the unit cell in Figure 2(A). Crucially, changes to the capacitance or inductance will modify the resonance and since the local dielectric environment is easily accessed, materials integration with SRRs provides a powerful approach to realize active metamaterials.

#### 2.1.2 All-dielectric metamaterials

Metamaterials and metasurfaces can also be formed solely from dielectric materials [57–59], (Figure 2C and E), which offers some advantages for various applications including zero Ohmic loss and higher temperature operation. The interaction between the all-dielectric structures and electromagnetic radiation has been well studied using effective medium theory [60], temporal coupled mode theory [61], equivalent surface current model [62], and Mie-type scattering theory [63]. Another theory for periodic systems, such as photonic crystals, includes Floquet–Bloch theory [64]. Here, we would like to show the resonant modes of the all-dielectric metamaterials using waveguide theory, which is directly derived from Maxwell’s Equations [65]. All-dielectric metamaterials (AMDs) utilize hybrid electric (EH) and hybrid magnetic (HE) waveguide modes that occur in various dielectric shapes, including spheres, cylinders, cubes, etc. Similar to metal-based metamaterials, the magnetic and electric response of ADMs may be tuned in $\omega_{\text{p}}^{2}$ , *ω*_0_ with the geometry of the unit-cell. For example, for cylinders (Figure 2C) the height *h* primarily modifies the magnetic *ω*_0_ while the radius *r* primarily modifies the electric *ω*_0_, although both modes have a weak dependence on other parameters [61].

Through treating the unit-cell of an ADM as a single dielectric resonator, we may solve the wave equation considering the boundary conditions of – for example – a cylindrical resonator (see Figure 2C). We are interested in the case when the electromagnetic wave is propagating with the k-vector parallel to the cylindrical axis – the *z*-axis. Two primary (dominant) modes result, a quasi-TE mode and a quasi-TM mode, where TE stands for transverse electric and TM stands for transverse magnetic, i.e. both are approximately transverse to **k**. These modes are hybrid waveguide modes, and thus the electric and magnetic fields are not strictly transverse to the propagation direction. Rather, for the quasi-TM mode, the *H*_*z*_ component is much weaker than the *E*_*z*_ component, i.e. *H*_*z*_/*E*_*z*_ ≪ 1, and the mode is denoted as an HE mode. Likewise, for the quasi-TE mode *E*_*z*_/*H*_*z*_ ≪ 1, which is termed an EH mode. The subscripts, *l,m,n* are used to denote the number of oscillations of the fields along the *r*, *ϕ*, *z*-directions respectively, e.g., HE_*lmn*_. Thus, the first order electric mode is EH_111_ and the first order magnetic mode HE_111_.

##### 2.1.2.1 EH Mode

The quasi-TE mode (EH mode) gives rise to an electrically resonant response and is similar to that yielded by an electric ring resonator (LC resonator) [53, 66]. We next show the results of waveguide theory, which gives the dependence of the mode on geometry [65, 67, 68]. Through application of the boundary conditions and matching fields inside and outside the resonator yields, tan(*k*_*z*_ *h*)/2 = (*k*_*z*0_)/(*k*_*z*_), where *k*_*r*_ is the radial wave vector component in the cylinder, *k*_*z*_ is the *z* component of the wave vector in the cylinder, while *k*_*z*0_ describes the *z* component of wave vector in air. The wave vectors satisfy $k_{z}^{2}=k_{0}^{2}\epsilon_{1r}-k_{r}^{2}$ and $k_{z0}^{2}=k_{r}^{2}-k_{0}^{2}$ , where *k*_0_ = *ω*_0_/*c* is the wavenumber in air and $n=\sqrt{\epsilon_{r}}$ is the index where *ϵ*_*r*_ is the real part of relative permittivity of the cylinder. In terms of free-space wavelength and index we find,

(2) $$h=\frac{\lambda_{0}}{\pi }\tan^{-1}\left(\sqrt{n^{2}-2}\right)$$

Matching the fields for the EH mode in the radial direction gives, *J*_1,1_(*k*_*r*_ *r*) = 0. The first non-trivial root of the Bessel function is 3.8317, which can be solved for the radius and expressed in terms of wavelength and index of refraction *n* as,

(3) $$r=\frac{3.83}{2\pi }\frac{\lambda_{0}}{\sqrt{n^{2}-1}}$$

Equations (2) and (3) are the final solutions of the EH mode in terms of determining the geometrical parameters to achieve a resonant response for a given *λ*_0_.

##### 2.1.2.2 HE Mode

Following the same approach, the quasi-TM (HE mode) is described by,

(4) $$h=\frac{\lambda_{0}}{2n}$$

Boundary conditions for the HE mode applied to the circular surface, i.e. matching the fields inside and outside the resonator, result in,

(5) $$\begin{array}{ll} & \left[\frac{J_{1}^{\prime }\left(u\right)}{uJ_{1}\left(u\right)}+\frac{K_{1}^{\prime }\left(v\right)}{vK_{1}\left(v\right)}\right]\\ & \;\times \left[k_{0}^{2}\epsilon_{1r}\frac{j_{1}^{\prime }\left(u\right)}{uJ_{1}\left(u\right)}+k_{0}^{2}\frac{K_{1}^{\prime }\left(v\right)}{vK_{1}\left(v\right)}\right]=k_{z}^{2}{\left(\frac{1}{u^{2}}+\frac{1}{v^{2}}\right)}^{2} \end{array}$$

where *u* = *k*_*r*_ *r*, *v* = *k*_*r*0_ *r*, and *k*_*r*_ is the radial wave vector in the dielectric cylinder, *k*_*z*_ is the wave vector along the cylinder axis in *z* direction, *k*_*r*_ is the radial wave vector in the dielectric cylinder, and *k*_*r*0_ is the radial wave vector in air, *J*_1_(*u*) is the first order Bessel function of the first kind, and *K*_1_(*u*) is the first order modified Hankel function. The wave vectors *k*_*r*_, *k*_*r*0_, and *k*_*z*_ satisfy $k_{r}^{2}=k_{0}^{2}\epsilon_{1r}-k_{z}^{2}$ and $k_{r0}^{2}=k_{z}^{2}-k_{0}^{2}$ . The solution to the HE mode given by Eq. (4) may be solved directly to find the required height *h* that would give the resonance frequency, and Eq. (5) is transcendental and may be solved graphically to give the radius *r*.

**Figure 3:**
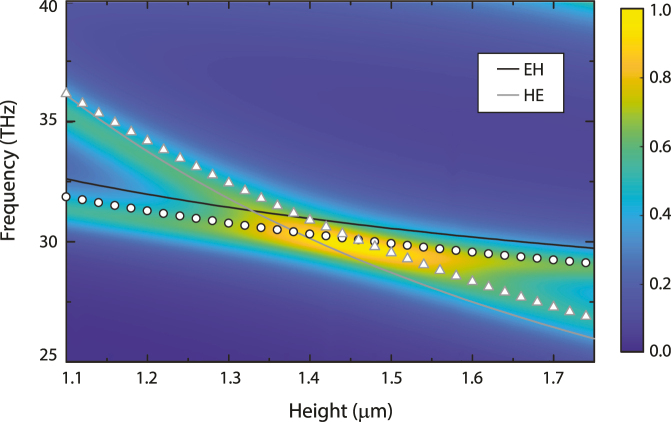
Dependence of the modes supported by an all-dielectric metamaterial as a function of height and frequency. The color plot shows the absorptivity, the open symbols are from Eigenvalue simulations, and the solid curves are from the theory presented in Eqs. (2)–(5). Black colors are for the EH_111_ mode, and gray colors for HE_111_.

Using Eqs. (2)–(5) we solve for the resonant frequency of both the EH_111_ and HE_111_ modes as a function of the height of a cylindrical all-dielectric metasurface, with radius *r* = 1.75 μm, a periodicity of *p* = 6.8 μm, and fashioned from lossy germanium with a real permittivity of *ϵ*_*r*_ = 16.0 and a loss tangent of tan *δ* = 0.035. The theoretical results are plotted in Figure 3 as the solid black (EH_111_) and solid gray (HE_111_) curves. The color plot shows the height and frequency dependence of the simulated absorptivity – calculated as *A*(*ω*) = 1 − |*S*(*ω*)_11_|^2^ − |*S*(*ω*)_21_|^2^, with values indicated by the colormap, and where *S*(*ω*)_*ij*_ are the elements of scattering matrix for a 2-port system [69]. Results from eigenvalue simulations are shown as the open black symbols (EH_111_) and open gray symbols (HE_111_). Excellent agreement between the theoretical curves Eqs. (2)–(5) and numerical simulations are evident, and we find a maximum difference of 2.4% and 3.1% for the EH and HE modes, respectively.

We note that, unlike metal-based metamaterials, ADMs do not possess internal capacitive regions for field localization, and can exhibit significant evanescent field values outside the geometrical bounds. ADM unit-cells may also be used to tesselate a surface in an arbitrary fashion similar to that achieved with metal-based metamaterials (Figure 2E). However, the aforementioned evanescent fields can lead to significant nearest neighbor interactions. Further, ADMs resonators are only marginally subwavelength, and surface modes related to the periodicity are not restricted to nearest neighbors, and may persist over several spatial periods (i.e. the modes are not confined to a unit cell). This presents additional challenges in creating active ADMs, but as the examples in this review show, it is still possible since the local dielectric environment is accessible and is therefore amenable to materials integration. The properties of the dielectric resonators can also be modified using, for example, optical excitation which will modify the resonant response.

### 2.2 Other APM

While not the focus of this review, we briefly mention other common APMs as there is conceptual overlap with metallic and dielectric metamaterials with ample opportunity for active and tunable control. For example, metallic nano-sized particles may support light driven coherent electron oscillations in the visible regime [6, 70, 71]. These resonances are called localized surface plasmon resonance (LSPR), with the resonant frequency governed by the size (geometry), composition, embedding dielectric environment, and inter-particle spacing. Noble metals are often used, including Ag and Au, which enable subwavelength confinement of light.

Another common APM are photonic crystals (PCs) – also termed photonic band gap (PBG), or electromagnetic band gap (EBG) materials. Photonic crystals consist of inhomogeneous periodic objects or homogeneous dielectric with spatially varying permittivity that either prevent (band-gap) or permit (defect state) the propagation of electromagnetic waves at a particular wavelength, or over a band of frequencies [7, 36]. A common grand challenge goal of PCs is to design band-gaps or pass-bands for all incident angles and polarization states of incident light [33]. The basic physical phenomenon underlying operation of PCs is that of diffraction, and thus the periodicity of a PCs is of the order of the operational wavelength. Photonic crystals may be fashioned to operate in one, two, or three dimensions [72, 73].

With this brief introduction to APM, we next discuss various active materials that can be integrated with metamaterials and external stimuli that can be employed to modify the properties of the active materials and, thereby, the metamaterial electromagnetic response.

## 3 External stimuli and materials

As described in the previous section, electromagnetic metamaterials and metasurfaces may be fashioned from metallic base materials or from dielectric materials. We make a distinction between the metallic metamaterials (MMs) and ADMs because the principles underlying their dynamic properties differ. In MMs for instance, the electric field is strongly enhanced to sub-wavelength volumes within the split-gap *g* of the structure, as shown in Figure 2(A), near the resonance frequency *ω*_0_. We define the field enhancement to be the ratio of the maximum electric field strength in the gap *E*_g_ to the value of the incident electric field strength *E*_i_, i.e. *F* ≡ *E*_g_/*E*_i_. In plasmonic metamaterials, the limit of *F* was found in a semi-classical model to be determined by the Thomas–Fermi screening length *λ*_F_ = *v*_TF_/*ω*_p_, where *v*_F_ is the Fermi velocity, and *ω*_p_ is the plasma frequency of the metal constituting the MM [74]. Experiments conducted in the visible and near infrared regimes with plasmonic gold spheres spaced angstrom distances above a conducting ground plane found *F* ∼ 10^2^ − 10^3^ [74]. The permittivity (*ϵ*_*g*_) of materials placed within the gap (along with the geometry), determines the resonance frequency *ω*_0_ = (*LC*)^−1/2^, where *C* = *ϵ*_*g*_ *A*/*g* and *A* is the area of the gap region of the metamaterial. Thus, if the field is strongly enhanced within the sub-wavelength gap region – volume of *V* = *A* × *g*, then *ω*_0_ is primarily determined by *ϵ*_*g*_. External stimuli that are capable of modifying *ϵ*_*g*_ may therefore be used to enable dynamical properties in metal-based metamaterials.

All-dielectric metamaterials support hybrid waveguide modes as described in Section 2.1.2 (also described as Mie resonances) which occupy approximately the same volume as the ADM resonator itself. Since ADMs are only marginally sub-wavelength, the enhancement possible is significantly reduced compared to metal-based metamaterials. However, since the entire ADM may be fashioned from materials amenable to changes in material properties, e.g., permittivity, alternative means of achieving dynamic properties become possible. That is, by fashioning ADMs out of semiconducting, superconducting, phase change, and other similar materials, dynamic properties can be obtained. Specifically, Eqs. (2)–(5) show the dependence of the height *h* and radius *r* necessary to achieve two resonances of opposite symmetry, and their relation to the index of refraction $n=\sqrt{\epsilon_{r}\mu_{r}}$ . Also, ADMs possess an evanescent component that is nonzero outside of the ADM surface. Thus, one may make ADMs with small spacing between unit-cells in order to increase *F*.

From this discussion, it is evident that control of the local electromagnetic fields in MMs or ADMs provides the means to create active metamaterial devices. Myriad routes to achieve this are possible both in terms of the stimuli and materials that are used. We first discuss the primary stimulus methods, followed by detailed examples of materials that have been integrated with metamaterials.

### 3.1 External stimuli

**Figure 4:**
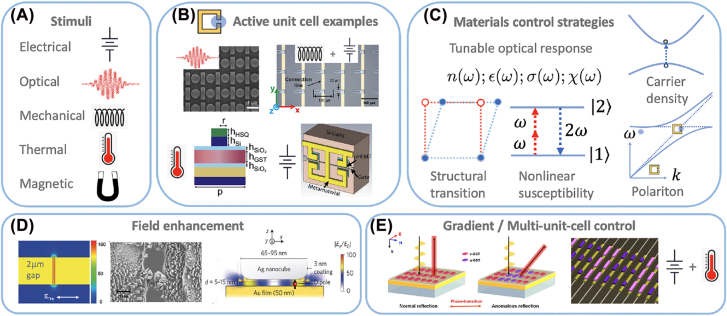
(A) Various stimuli to realize active metamaterial control. (B) Examples of unit cells amenable to active/nonlinear control. Upper left: Si bar-disk resonator which exhibits a field enhanced nonlinear response leading to an increase in the efficiency of high harmonic generation. Reprinted from [76], Copyright 2018, Nature Publishing Group. Upper right: a cantilever controlled metasurface to realize surface wave switching in the terahertz range. Reprinted from [77], Copyright 2022, Optica. Lower left: a near-IR plasmonic metamaterial where thermal control is achieved using GST. Reprinted from [78], Copyright 2021, ACS. Lower right: terahertz split ring resonator with HEMT integration for high frequency modulation. Reprinted from [79], Copyright 2011, Optica. (C) Materials integration enables control through of the dielectric response. This includes structural control, carrier doping, nonlinear effects, and polaritonic tuning. (D) Metasurface gap structures provide large on-resonance electric field enhancement and localization (left simulation) which can play an important role in nonlinear active metamaterials. (D) Middle panel shows an image of metamaterial gap structure demonstrating electromigration due to the large electric field concentration. Left and center image: Reprinted from [80], Copyright 2015, AIP. Right image: Reprinted from [81], Copyright 2014, Nature Publishing Group. (E) Active control can also be realized at the unit-cell level to tune the gradient/phase response of metasurfaces – the example depicted here is current-based thermal control of GST.Reprinted from [87], Copyright 2021, Wiley.

Figure 4 presents an overview of important aspects of external stimuli and materials integration to realize active metamaterials. A number of different types of external stimuli may be used to achieve active and tunable nanophotonic metamaterial responses. This includes (Figure 4A) electrical, optical (including nonlinear optics), mechanical, thermal, and magnetic approaches. Real-time tunable and active control ultimately arises from use of these external stimuli to modify the local electric and magnetic fields in strategic locations within the metamaterial or metasurface unit-cell. In the case of mechanical actuation, the local fields are altered by physical motion of constituents within the unit-cell.

Figure 4(B) presents several examples of unit cells that exhibit active responses (each example is labeled with a corresponding symbol in Figure 4(A) indicating the nature of the stimulus). The upper left is an example of an ADM fabricated from Si on a sapphire substrate that is resonant in the mid-infrared. These bar antenna disk resonator structures exhibit an electromagnetic induced transparency response with enhanced local fields which can, in turn, lead to an enhanced nonlinear response including high-harmonic generation [75, 76]. This is an example where the material from which the metasurface is fabricated also serves to create a dynamic nonlinear response. The upper right panel (Figure 3(B)) is a cantilever actuated metasurface that can enable terahertz surface wave switching and is an example utilizing electrical stimulation to induce a mechanical response that modifies the electromagnetic properties [77]. The lower left panel shows a cross-section of a multi-layer unit cell that integrates the phase change material germanium antimony telluride (Ge_2_Sb_2_Te_5_ or GST) into a Si-Au plasmonic structure to enable thermal control of the electromagnetic response arising from changes in the refractive index of GST that manifest with the degree of crystallinity [78]. Finally, the lower right depicts a metallic SRR where high-electron mobility transistors have been integrated into the capacitive gaps for high-speed modulation of the resonant metamaterial response [79]. These are but a few examples highlighting the diversity of materials amenable to integration with metamaterials - other examples will be presented below in greater detail.

The methods of stimuli in Figure 4(A) provide the means to alter the optical and electronic properties of the materials that are integrated into the metamaterial structures via changes of the local dielectric response described by the frequency-dependent permittivity *ϵ*(*ω*) = *ϵ*_1_(*ω*) + *iϵ*_2_(*ω*) or, equivalently, the optical conductivity *σ*(*ω*) = *σ*_1_(*ω*) + *iσ*_2_(*ω*), refractive index *n*(*ω*) = *n*_1_(*ω*) + *in*_2_(*ω*), or susceptibility *χ*(*ω*) = *χ*_1_(*ω*) + *iχ*_2_(*ω*). The choice of which response function to use is a matter of convenience for a given problem or application. Figure 4(C) depicts routes through which *ϵ*(*ω*) can be modified (in principle by any of the stimuli in Figure 4(A)). This includes: (i) structural changes as in the case of liquid crystals or phase change materials; (ii) carrier density control in semiconductors; (iii) modification of the nonlinear response (nonresonant or resonant). This case is interesting because the local field enhancement of the metamaterials can lower the threshold for the onset of a nonlinear response. As mentioned in the introduction to this section, the field enhancement can be as large as 10^2^–10^3^. Figure 4(D) shows examples of the large field enhancements that are possible at terahertz frequencies and at visible frequencies [80, 81]. Since nonlinear responses scale as the electric field to some power, the effects can be quite large. A final case depicted in Figure 4(C) is that of polariton [82]. In most cases, the resonances of the materials that are integrated into the metamaterials do not coincide with the resonance of the metallic or dielectric resonators. However, mode-splitting and other strong coupling effects arise when the material and metamaterial resonances overlap offering an additional route for active control [83–86]. Finally, we mention that active control can extend to more complex unit cells (a conceptual example using current-induced thermal control of GST is depicted in 4(E)) where, in principle, the actuation can be controlled at the unit cell level to achieve subwavelength gradient control of the electromagnetic response [87].

#### 3.1.1 Electronic

We briefly consider general aspects of the stimuli listed in Figure 4(A). Electric fields are particularly important, as it is relatively easy to apply a potential difference which enables control of the carrier density, current, or to generate electrostatic forces for mechanical actuation and geometrical control. For metallic metamaterials, the structure can serve as an electrode. Electric fields enable precise control of the carrier density in semiconductors or 2D materials which can effectively shunt capacitive regions of a metamaterial, thereby modifying the resonant electromagnetic response [88, 89]. Or electric fields can reorient the molecules comprising a liquid crystal as a route to modify the metamaterial response [90–92]. Other examples include current induced heating to drive phase changes in materials or mechanical actuation to drive changes in the geometry or orientation of a metamaterial [93–95]. Electric field control of metamaterials can range from static to GHz or beyond, depending on the mechanism and materials used. In addition to input–output control (Figure 1(A)), electric fields also enable transduction for novel light detection [96]. Finally, at microwave frequencies, varactor-based active metamaterials have been demonstrated [97–99].

#### 3.1.2 Optical

Optical stimulation of metamaterials also offers numerous routes for active MM control. This modality can utilize simpler metamaterial structures in comparison to electrical control, though at the expense of requiring an excitation source and associated optical components. Active modalities include carrier density control via above-gap excitation in semiconductors [100]. Optical excitation also provides ultrafast control dictated by the carrier dynamics of the materials that are used to craft the metamaterials. It is even possible to achieve control at the ps or sub-ps level [101]. Sub-gap excitation (e.g., with terahertz or mid-IR frequencies) enables novel control where, for example, the local field enhancement lowers the effective threshold for Zener tunneling, impact ionization, or dynamical Franz-Keldysh effects [102–104]. Beyond carrier density control, optically induced heating (for example, carrier relaxation via phonon emission) can be used with phase-change materials to tune the response of MM, analogous to current induced heating with electric field control [105, 106]. One of the most prolific areas of active MMs is nonlinear control where, as mentioned above, the local field enhancement (in metallic or ADM metamaterials) lowers the threshold for phenomena ranging from harmonic generation to bistability [107, 108]. Importantly, the field enhancement can overcome the volumetric reduction of the nonlinear material (i.e. the active nonlinear region is a fraction of the unit cell volume) such that the overall effect is larger than for a bulk nonlinear material [109, 110]. Of course, this comes with the additional benefits engendered by the metamaterial and allows for entirely novel effects such as nonlinear holography [111].

#### 3.1.3 Thermal

Thermal control of metamaterials typically employs materials that exhibit temperature dependent phase changes which includes structural and/or electronic changes. This includes materials such as GST discussed above, materials that exhibit insulator-to-metal transitions (with VO_2_ by far the dominant example explored to date for metamaterial integration), and superconductors which are interesting as a low-loss material (in the superconducting state), but also in terms of the novel physics associated with superconducting tunneling (e.g., Josephson junctions) [112–117]. Temperature tuning is slow (but sufficient for some applications such as for camouflage [112]) but in combination with optical or electrical stimulus provides a powerful approach to realize large changes in the electrodynamic response of metamaterials. For example, low power optical excitation can quench a superconductor on a picosecond timescale with, depending on the magnitude of the superconducting gap, recovery times ranging from ps to ns [118].

#### 3.1.4 Mechanical

Mechanical control is similar to thermal control in the sense that it is usually accomplished in combination with additional stimulus such as electrical actuation in micro-electromechanical systems (MEMS) as discussed in more detail in Section 3.2.5. However, applied strain using elastic polymers has been used to demonstrate metamaterial tuning [119–121]. Thermo-mechanical tuning is also possible, for controlled motion of a cantilever comprised to two materials with different thermal expansion coefficients [93, 122, 123]. There are also interesting possibilities for novel mechanical tuning in metamaterial nanostructures where optical excitation can drive coherent acoustic modes that subsequently modify the electrodynamic response on a picosecond timescale [124, 125].

#### 3.1.5 Magnetic

Finally, we mention magnetic control. Metamaterials and related plasmonic structures have been used to control the magnetic properties of materials with which they are integrated [126]. However, the opposite has also been achieved where modification of the magnetic properties tunes the resultant metamaterial properties [127, 128]. This includes magnetization switching of a magneto-plasmonic response, and magnetic switching of optical-chirality at visible and near-IR wavelengths [129–131]. Moreover, using magnetic fields to tune cyclotron resonance is 2DEGS or high mobility materials such as InAs are of interest for fundamental and applied metamaterials control at terahertz frequencies [84, 132]. MEMS based metamaterials have used magnetic fields arising from currents in adjacent structure to provide mechanical motion and reconfigurability [133].

Clearly, there are enormously rich possibilities using a host of external stimuli for active control of metamaterials spanning from the microwave through the visible. In the following section, we provide a few in-depth examples of active metamaterials control.

### 3.2 Materials

In this section, various materials and representative metamaterials integration examples are presented. This includes semiconductors, two-dimensional materials, transparent conductive oxides, liquid crystals, and micro-electro-mechanical systems. There are numerous materials systems which are compatible with metamaterials and which may be used to achieve dynamic and active properties. Although an exhaustive list of materials and representative works is beyond the scope of this manuscript, we list several review papers which cover various approaches and materials in order to aid the interested reader [22], [23], [24, 26, 27, 29]. We also make reference to an important recent work (see Table 1 of [29]) which presented an excellent table summarizing modulation mechanisms, active components, external stimuli, operational range, modulation depth, and advantages and limitations of methods to achieve dynamic responses, and therefore we do not reproduce this information here.

#### 3.2.1 Semiconductors

Semiconductors are materials which may achieve variable electrical conductivity values which lie in-between insulators (dielectrics) and conductors (metals). Silicon (Si) is an elemental semiconductor and the second most abundant element on earth. As such, it is a highly utilized semiconductor in photonic devices. Seminconductors which consist of two or more different elemental groups are termed compound semiconductors, and some examples are III–V, II–VI, and IV–IV groups. For example, common Group III–V compound semiconductors include: gallium–arsenide (GaAs), gallium–nitride (GaN), indium–phosphide (InP), and aluminium–gallium–indium–phosphide (AlGaInP). Other common compound semiconductors include Group II–VI (ZnS, ZnSe), and Group IV–IV (SiC, SiGe). Key features of compound semiconductors include direct energy bandgaps, high electron mobilities, and high breakdown electric fields. Typical semiconductor bandgaps range from ∼0.25 eV (PbSe) to 6 eV for aluminum nitride (AlN).

Metamaterials combined with semiconductors can exhibit dynamical electromagnetic properties, with two basic approaches to dope carriers in semiconductors, i.e. optical excitation or electrical bias. An early demonstration of optical photodoping of metamaterials was carried out at terahertz frequencies, where an electric LC (ELC) resonator metamaterial on a GaAs substrate was demonstrated [100]. A 10 mW ultrafast pulse at 800 nm wavelength was incident on the GaAs substrate, generating a carrier density of approximately *n* = 4 × 10^16^ cm^−3^. It was shown that this doping density is sufficient to fully short the gap of the ELC resonator, thus significantly weakening the resonance. More recently, optical photodoping has been widely utilized to modulation the metasurface properties, including resonance frequency [134, 135], amplitude [136, 137], phase [138], chirality [139, 140], effective permeability [141], quality factor [142, 143], and polarization [144].

In a different mode of operation, where the wavelength of pump light and the metamaterial resonance wavelength are the same, the metasurface field enhancement can further boost the electric field in the resonator and/or semiconductor. For example in the optical range, an optical pulse of approximately 100 fs – in concert with the enhanced metasurface electric field – enables harmonic generation within the semiconductor [76, 145], [146], [147]. At longer infrared wavelengths, intense high-field terahertz pulses can last over one picosecond, such that carriers in the semiconductor are ballistically accelerated, acquiring a kinetic energy greater than several electron volts. As a result, carrier dynamics can be induced including impact ionization, intervalley scattering, and the Poole–Frankel effect, in addition to others [102, 148], [149], [150]. More generally, the theoretical limit of modulation speed using photodoping methods is determined by the recombination lifetime of the carriers, which varies from microseconds to picoseconds depending on the properties of materials [101]. Optical excitation methods can easily generate carriers where number densities are increased by several orders of magnitude. However, such techniques cannot be easily implemented for practical applications, especially for circumstances requiring spatial addressing.

An all-electronic approach for control of semiconducting metamaterials is an attractive proposition. Metal-based metamaterials may be relatively easily spatially addressed, allowing for individual control of pixels at various sizes down to the unit-cell level. Further, metamaterials are multifunctional which permits biasing with electrical control lines that form part of the patterned metal itself [88]. Similar to the optical photodoping case described above, if a semiconductor is material doped such that its carrier density *n* is large enough, it can exhibit conductive properties at a frequency *ω*_0_. If this doped semiconductor is placed within the gap of a metamaterial, it may result in a short circuit of the capacitive response, thereby quenching the resonance. However, application of an electric field to the metamaterial may be used to deplete (repel) charges in the semiconductor, away from the gap, thereby tuning it into a dielectric, and bringing back the resonance at *ω*_0_.

#### 3.2.2 Two dimensional materials

Two-dimensional (2D) materials have emerged as a promising alternative to silicon due to their extraordinary carrier dynamics even for atomically thin channel thickness. At the same time, their exotic optical properties have also enabled 2D materials as a versatile platform for photonic applications. Graphene is the first discovered 2D material with an atomic thickness of only 3.45 Å and zero bandgap. Followed by this inspiring discovery, various new 2D materials have been investigated, including transition metal dichalcogenides (TMDCs) such as MoS_2_, WS_2_, MoSe_2_, and WSe_2_, hexagonal boron nitride (h-BN), black phosphorous (BP), and mono-elemental compounds (Xenes), etc. Through the hybridization of 2D materials with artificial electromagnetic materials, unprecedented dynamic properties not easily achieved in other materials have been demonstrated. This includes high-frequency modulation [154], highly efficient modulation depth [155], and photoluminescence from extremely small mode volumes, as but a few examples [156].

**Figure 5:**
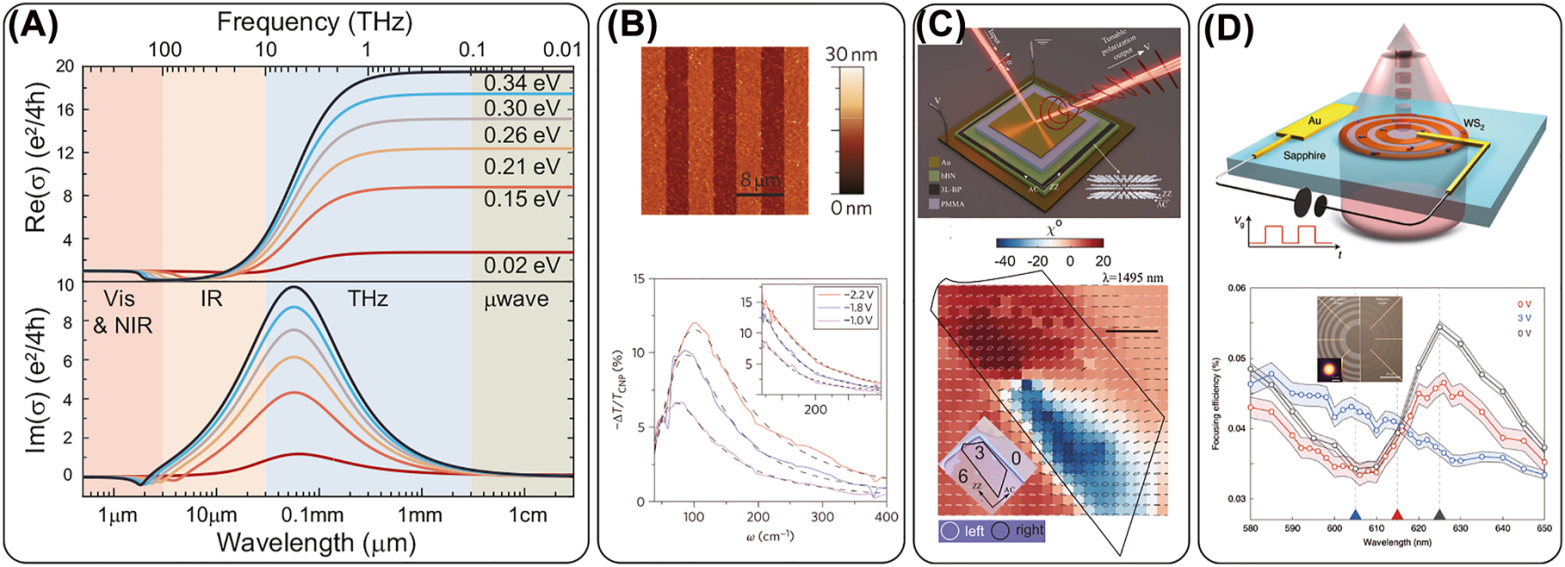
(A) Bias dependent conductivity of graphene over the electromagnetic spectrum from microwave to visible wavelengths. The conductivity is calculated with an assumption of 50 nm Al_2_O_3_ underneath the graphene layer and an initial doping of 5 × 10^10^ cm^−2^ from defects. (B) Graphene based plasmonic metasurfaces. Top: AFM image of a fabricated micro-ribbon array of graphene (light colors) on top of silicon dioxide (dark colors), each of 4 μm width. Scale bar is 8 μm. Bottom: Plasmonic resonance varied with electrical gating. The inset shows the response with polarization along the ribbons. Reprinted from [151], Copyright 2011, Springer Nature. (C) Polarization conversion on a tri-layer black phosphorus. Top: Schematic of an electrically tunable metasurface with polarization conversion. Bottom: A spatial color map of ellipticity at 1495 nm with reflected polarization ellipses varying from point to point. The inset shows the optical image of the device. The outlined region covers the tri-layer BP. Reprinted from [152], Copyright 2021 AAAS. (D) Top: Schematic of an electrically tunable WS_2_ zone plate lens. Bottom: Focusing efficiency spectra of the lens under different bias. The shaded area shows the error bar corresponding to one standard deviation. The inset shows the optical microscope image of the center of a fabricated lens (right) and the designed WS_2_ pattern. Reprinted from [153], Copyright 2020, Springer Nature.

*Graphene* has shown enormous potential for photonic and optoelectronic applications. Due to the linear dispersion of massless Dirac fermions, graphene exhibits unique properties with very high carrier mobility reaching a value of 3.50 × 10^5^ cm^2^ V^−1^ s^−1^, a broadband optical response and wide range of optical conductivity via electrostatic tuning, or chemical or optical doping. The optical conductance of a graphene monolayer in the visible range is frequency independent with a value of *G*_0_ = e^2^/4*ℏ*, leading to a constant absorption of 2.3%. The light-matter interaction in graphene monolayers involves two process: interband transition and intraband transition, described using the following equation for the optical conductivity [157, 158]:

(6) $$\begin{array}{ll} \sigma \left(\omega \right) & =\frac{{\text{e}}^{2}}{4\hbar }\left[\frac{1}{2}+\frac{1}{\pi }\arctan\left(\frac{\hbar \omega -2E_{\text{F}}}{2k_{\text{B}}T}\right)\right.\\ & \;-\left.\frac{i}{2\pi }\ln\frac{{\left(\hbar \omega +2E_{\text{F}}\right)}^{2}}{{\left(\hbar \omega -2E_{\text{F}}\right)}^{2}+4{\left(k_{\text{B}}T\right)}^{2}}\right]\\ & \;+\frac{i2{\text{e}}^{2}k_{\text{B}}T}{\pi \hbar^{2}\left(\omega +i\omega \tau^{-1}\right)}\ln\left[2\;\cosh\left(\frac{E_{\text{F}}}{2k_{\text{B}}T}\right)\right] \end{array}$$

where *k*_B_ is the Boltzmann constant, *T* is the temperature, *ω* is the frequency, *τ* is the carrier relaxation time, and E_F _ is the Fermi level. The first term of Eq. (6) accounts for interband transitions, which mainly dominates in the range of optical and infrared frequencies. The second term describes intraband transitions, which is relevant for terahertz and microwave frequencies behaving as a Drude-like semiconductor. Electrostatic or optical doping of carriers changes the Fermi energy in graphene, thereby modulating the optical conductivity. Figure 5(A) shows the optical conductivity spectra for various gating voltages for a graphene monolayer with a scattering time of 30 fs, and initial doping of 5 × 10^10^ cm^−2^. Clearly, the conductivity of graphene can be changed from microwave through near-infrared frequencies, which is a much larger range than other semiconductors or transparent conductive oxides, which only provide effective conductivity tuning over a fraction of the spectral range [159, 160].

Because of the facile tuning of the Fermi level, graphene offers tremendous potential for applications to control light–matter interactions from infrared to microwave range frequencies. In the microwave and terahertz range, graphene can be basically considered as an extremely thin impedance surface with a tunable resistance [161]. Such a large conductivity modulation also works well in the terahertz range with significant modulation on the transmission [12, 89], phase [162], polarization [163], Brewster angle modulation [164], etc. In the infrared range, both the real and imaginary parts of the conductivity exhibit significant change with the tuning of the Fermi level. The change of real part of permittivity mainly contributes to the resonant wavelength shift while the modulation of the imaginary part can be used to control the resonance bandwidth. Although graphene exhibits a considerably smaller conductivity modulation amplitude in the infrared region compared to the far-infrared and microwave regions, remarkable modulation of the phase, reflection, emissivity, and Fano resonance, have nonetheless been experimentally demonstrated [154, 165], [166], [167], [168], [169], [170], [171], [172]. In addition, because of the intraband transition contribution to the surface conductivity in the infrared and terahertz range is similar to that of plasmonic metals in the visible range, graphene has also been considered as a new platform for surface plasmonic waveguiding with intriguing properties, such as extreme light confinement and active modulation of the plasmonic response as shown in Figure 5B [151]. The high carrier mobility has enabled a modulation frequency of dynamic graphene metasurfaces up to ∼20 GHz [154].

*Black Phosphorous* (BP) is a group-V layered material with very high carrier mobility, reaching about 5200 and 4.50 × 10^4^ cm^2^ V^−1^ s^−1^ at room temperature and cryogenic temperatures for a 4 nm layer, respectively [173]. BP exhibits a layer-number dependent direct bandgap varying from 0.3 to 2 eV, which is favorable for electronic transistors with on-off current ratio as large as 10^5^ (this is significantly better than graphene which possesses a very large dark current, as a zero-gap material). BP can be used from the infrared to visible range, for various photonic applications, such as photodetection and modulation. The other distinct feature of BP compared to graphene, is the asymmetric lattice structure, leading to anisotropic optical properties, including birefringence and an anisotropic extinction coefficient along each axis in armchair and zigzag directions. Therefore, modulation of the anisotropic properties could lead to interesting phenomena not attainable from other 2D materials, such as changing the polarization state through modulating the absorption of light in different directions. It has been shown that the anisotropic optical conductivity of BP can be achieved through electrostatic doping, optical doping and strain engineering [174–176]. The optical conductivity of BP can be tuned via changing the Fermi level. Similar to graphene, with an increase of the Fermi level, the absorption edge exhibits a blue shift due to the Pauli-blocking of intersubband transitions, also known as the Burstein–Moss effect. In addition, it is found that the bandgap of BP can be adjusted by a vertical electric field, known as the quantum-confined Stark effect, in which a strong electric field tilts the bands, effectively inducing a smaller bandgap. As shown in Figure 5(C), an electrically tunable polarization converter can be achieved by placing tri-layer BP into an hBN/BP/hBN/Au stacked Fabry–Pérot structure [152]. Through electron or hole doping in the BP layer, the absorption for the polarization along the armchair direction can be dynamically modulated, while the absorption polarized along the zigzag direction is barely changed. Consequently, judicious designs achieve linear to circular and cross-polarization conversion under electrostatic bias, promising a new route for nontrivial polarization control in electro-optic applications.

*Transition Metal Dichalcogenides (TMDCs)* 2D TMDCs represent another category of ultrathin semiconductor materials with a typical form of MX_2_, where M is a transition metal, such as Mo and W, and X is a chalcogen including S, Se or Te. Although TMDCs have been known for about one century, they have not drawn significant attention until recent advances on nanofabrication, characterization techniques, and graphene-related research. As TMDCs are thinned down to few- or monolayer sheets, their bandgap undergoes a indirect-to-direct transition with the valence band maximum and the conduction band minimum at the energy-degenerate points K and K′ in the Brillouin zone. Interestingly, the spin degeneracy at points K and K′ is lifted by the broken inversion (time-reversal) symmetry which thus causes a spin splitting of the electronic bands with a strong spin-valley locking. Such an exceptional feature enables access to spins and the valley degree of freedom through controlling the photon helicity, revealing tremendous potential for novel spintronic and valleytronic devices. To overcome the weak light–matter interaction of thin layers, plasmonic and all-dielectric metasurfaces have been adopted as an enhancer to boost the photoluminescence efficiency [156, 177], [178], [179]. and second-harmonic generation [180, 181] Through integration with chirality-dependent metasurfaces, such as in-plane chiral asymmetric gratings or metasurfaces with a Pancharantnam–Berry phase gradient, the valley-polarized excitons and the spin-valley dependent SHG at the exciton resonance can be spatially separated and subsequently steered along user-defined directions [182, 183]. In addition to direct coupling of TMDCs as passive devices, recent experiments have shown that a monolayer MoSe_2_ embedded in a van der Waals heterostructure with graphene as a top gate can be electrically tuned as an atomically thin mirror due to the electron–exciton interaction [184]. The electrical tuning of the exciton resonance can also be used for tuning the focusing efficiency of an ultrathin lens based on a patterned WS_2_/graphene heterostructure [153]. As shown in Figure 5(D), a WS_2_ monolayer is patterned into a zone plate with 202 concentric rings and electrochemically gated by an ionic liquid. Due to the screened electron–hole interaction from the excess electron density under a 3 V gate bias, the exciton resonances at 520 nm and 625 nm can be significantly suppressed. In this way, the focusing efficiency of the zone plate can be efficiently switched on and off.

#### 3.2.3 Transparent conductive oxides (TCOs)

TCOs, such as indium tin oxide (ITO), aluminum-doped zinc oxide (AZO), and gallium-doped zinc oxide (GZO), are oxide semiconductors with large bandgaps that can be heavily doped up to 10^22^ cm^−3^, which is close to the carrier concentration of metals. Because of the large DC conductivity, TCOs have been widely used as transparent electrodes in display panels. Further, these materials exhibit metal-like optical properties in the NIR range. Similar to semiconductors, the density of carriers can be modified by various external stimuli, such as optical pumping and electrical biasing, thereby leading to tunable optical properties in the NIR range, which is challenging to achieve using conventional semiconductors. Another distinct advantage of TCOs lies in the lower loss from lower carrier density compared to conventional metals. Therefore, TCOs have potential as alternative plasmonic materials in the infrared range, and also represent a new type of active material for dynamically tuning the response of metasurfaces.

**Figure 6:**
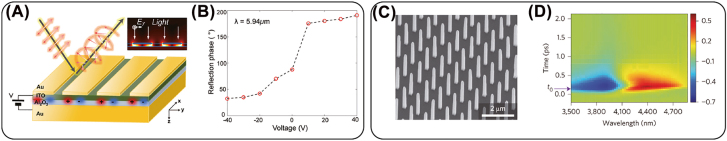
(A) Illustration of an electrically tunable metasurface for phase and polarization control using ITO thin film between two metal layers. The inset shows the distribution of electric field on resonance. (B) Phase control as a function of gating voltage at wavelength of 5.94 μm. (A) and (B) reprinted from [185], Copyright 2016 American Chemical Society. (C) SEM image of an ITO nanorod array (ITO-NRA) with 1 μm periodicity and 2.6 μm height. (D) Transient absorption measurement of the ITO ITO-NRA. The spectral map of the change of optical density (ΔOD) around the long-localized surface plasmon resonance (LSPR) measured by ultrafast NIR-pump-MIR-probe experiments. (C) and (D) reprinted from [186], Copyright 2016, Springer Nature.

Through application of a bias over a TCO thin film between two conductive layers, such as gating by structured metasurfaces and solid electrolyte, it has been observed that the carrier concentration within several nanometers at the metal-TCO interface can blue shift the plasma frequency [187–189]. Consequently, the permittivity for longer wavelengths, e.g. the mid-infrared region, the TCO functions as a lossy material, which is similar to doped silicon in the terahertz region. Through judicious design of the plasmonic structure with a controlled material loss rate, a 180*°* reflection phase modulation was demonstrated at 5.94 μm, as shown in Figure 6(A) and B [185]. Moreover, the blue shift of the plasma frequency can lead to a sign change from positive to negative for the permittivity in the NIR region (at the so-called epsilon-near-zero (ENZ) frequency). With such a sign change, plasmonic devices operating in this regime can show a pronounced reflection modulation of the amplitude and phase [190]. Applying 2.5 V between a gold ground plane and a patterned gold grating structure with a 20-nm ITO thin film, a 180*°* phase modulation and about 30% reflectance can be achieved near the ENZ region. Because of the small footprint of the device, the modulation speed, which is limited by the capacitance and device resistance, can reach 10 MHz [191].

In contrast to fast electrical modulation possible with a capacitive charging process, faster optical carrier excitation is possible. For example using ultrafast optical pulses to pump TCOs, such as ITO and the high-mobility indium-doped cadmium oxide (CdO), the thermalized hot electrons will populate higher energy levels in the conduction band, with a heavier effective mass due to the non-parabolicity of the bandstructure. As a result, the polarization selective resonance redshifts, showing a sub-picosecond response for phase and polarization switching [192–194]. As shown in Figure 6(C) and (D), the sub-bandgap pumping of ITO nanorod arrays enables an ultrafast plasmon modulation in the MIR range due to the distribution of hot-electrons higher into the nonparabolic conduction bands. A redshift of the localized surface plasmon resonance at both NIR and MIR frequencies was also observed [186].

TCOs are designed to achieve high transmission in the visible range. Specifically, the goal is to obtain a large screened plasma frequency $\hbar \omega_{\text{p}}=\hbar \left(e/\sqrt{\left(\;\right.}{\;\varepsilon }_{0}\varepsilon_{\text{r}\;}\left.\;\right)\right)\sqrt{n/m^{*}}$ , and a large electric DC conductivity with *σ*_DC_ = *enμ* = e^2^ *τ*(*n*/m*), where *ɛ*_0_, *ɛ*_r_ are the vacuum and relative permittivity, *e* is the electron charge, *n* the free carrier concentration, m* the effective carrier mass, μ the carrier mobility and *τ* the scattering time. Obviously, the ratio *n*/m* is the key factor for achieving transparency and high conductivity in designing TCOs. In conventional TCOs such as ITOs, the effort is to increase the dopant concentration in an oxide consisting of post-transition metals with small effective masses. An emerging strategy has been demonstrated in correlated metals such as SrVO_3_ and CaVO_3_. Due to the enhanced carrier effective mass from strong electron–electron interactions, the screened plasma energies exhibit values lower than 1.33 eV, which is much lower than the edge of the visible range, although the carrier concentration is higher than 2.2 × 10^22^ cm^−3^ [195]. Such a design method can also be considered for many other correlated metals, providing a new route for new TCOs and dynamic plasmonic devices.

#### 3.2.4 Liquid crystals

Liquid crystals (LCs) are anisotropic materials, consisting of a collection of dispersed elongated molecules, i.e. mesogens, oriented in a master solvent, either disorderly or ordered like a crystal. Of various LCs, twisted nematic LCs are the most general type, and used in commercial display products. LCs often exist in a rod-like molecular structure, where the long axis develops a dipole moment **p** = *q* **d**, from the separation of charge (*q*), which is proportional to the distance between them (*d*). Here **d** is the displacement vector which points from the negative to positive charge. Thus application of an external electric field results in a torque (*τ*) on the liquid crystal given by,

(7) τ=p×E

The resulting torque aligns the liquid crystals along the field lines, thereby altering its index of refraction $n\left(V\right)=\sqrt{\epsilon_{LC}\left(V\right)\mu }$ , where *ϵ*_*LC*_(*V*) is the voltage dependent dielectric constant of the liquid crystal.

Because of the rod-shaped molecules, an ensemble of these particles exhibits distinct polarizabilities as the induced dipole direction changes from parallel to the molecular long axis (optical axis) to perpendicular to the molecular long axis, thereby leading to optical anisotropy. For an external AC voltage applied across the nematic liquid crystal in a parallel plate configuration, the LC mesogens rotate with the induced dipole moment parallel to the static electric field. This occurs when the applied voltage is above a critical voltage known as the Fréedericksz threshold voltage *V*_*c*_ [196]. The rotation angle of the mesogens depends on the applied voltage. For a large enough voltage, the ordering of the mesogens enables a normally incidence plane wave to experience a refractive index perpendicular to the optical axis as *n*_o_ or *n*_⊥_. Similarly, the plane wave with polarization along the optical axis will experience a refractive index parallel to the optical axis as *n*_e_ or *n*_‖_. The birefringence of the LC is given as Δ*n* = *n*_e_ − *n*_o_ = *n*_‖_ − *n*_⊥_. In addition to electric tuning, LCs can also be controlled with other external stimuli, including heat and light.

**Figure 7:**
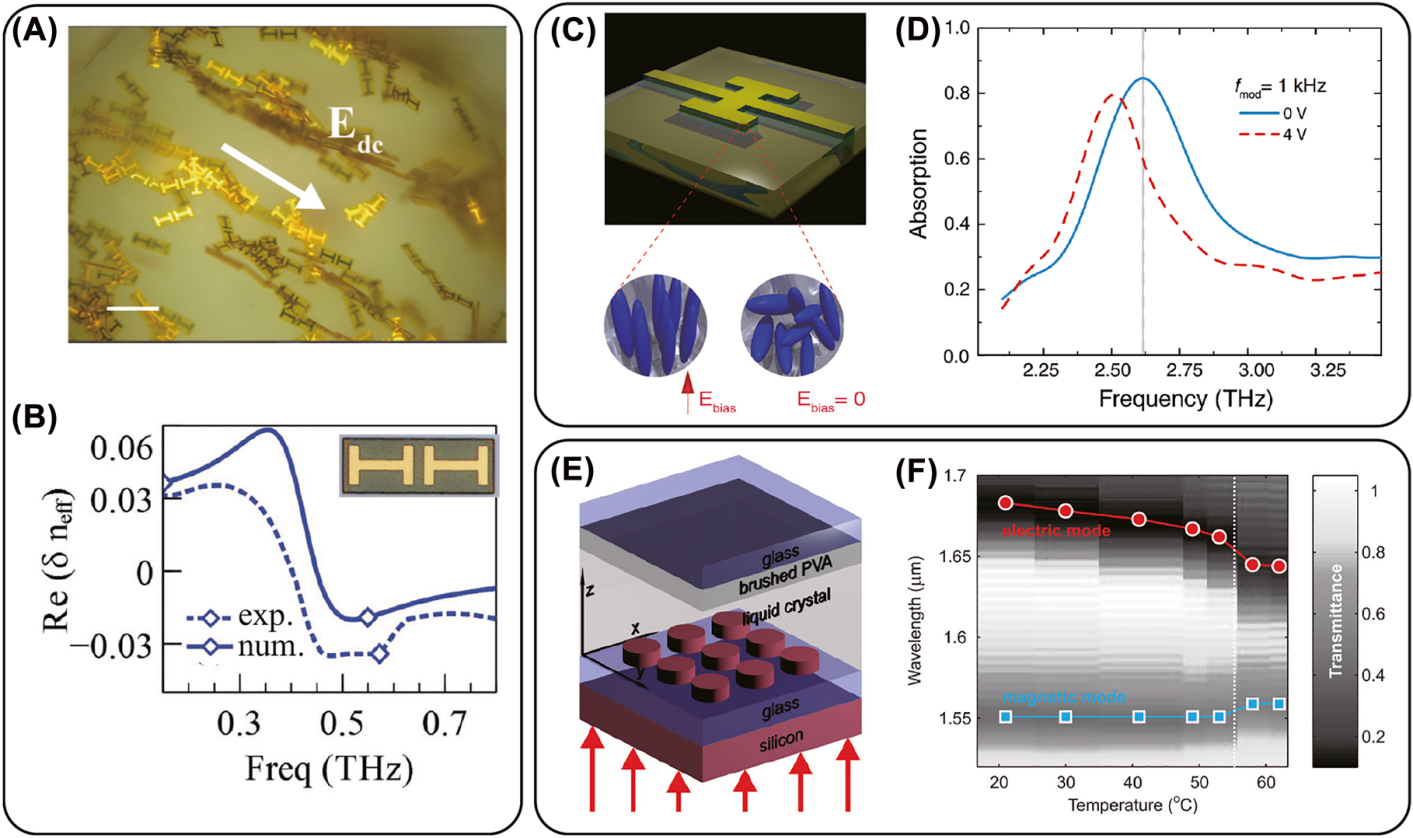
Liquid crystal based tunable metasurfaces. (A) Oriented in one direction of meta-mesogens mades of I-beam resonators under the application of bias electric field. (B) Effective index difference from measurements (dashed line) and simulations (solid line). (A) and (B) reprinted from [185], Copyright 2015 John Wiley & Sons. (C) Illustration of a tunable metasurface absorber with liquid crystal aligned with electric field bias and randomly without bias. (D) Measured absorption spectra for 0 V and 4 V at a modulation frequency of 1 kHz. The dashed line is centered at 2.62 THz. (C) and (D) reprinted from [90], Copyright 2013 American Physical Society. (E) Schematic of a tunable silicon nanodisk metasurface integrated with liquid crystal. (F) Measured transmittance spectra of the silicon nanodisk metasurface. The electric and magnetic resonances are plotted as red-dots and cyan squares, respectively. (E) and (F) reprinted from [199], Copyright 2015 American Chemical Society.

Most commercially available nematic LCs have a birefringence Δ*n* in the range 0.06–0.26 [197]. For example, the alkylcyanobiphenyl material 5CB shows a birefringence of 0.194 at visible frequencies. Although LCs are in common use at visible frequencies, they can also be used at other frequencies including microwave, terahertz and infrared. Table 1 lists the birefringence properties of two typical LCs (5CB and E7), from microwave to visible frequencies. The birefringent properties of many other LCs at microwave and millimeter-wave ranges are summarized in [198]. Although good results have been achieved with LCs made from nature materials, artificial liquid crystals can be made from subwavelength metamaterial resonators possessing anisotropic polarization dependence. As shown in Figure 7(A), elongated metamaterials are dispersed in a Paraffin oil. Under an external electric field, the meta-mesogens rotate and assemble into arrays of lines by electrophoretic forces. Because of anisotropic properties of the resonators, the ensemble exhibits large birefringence as shown in Figure 7(B) with the largest value of 0.06 for I-beam shape resonators.

**Table 1:** Birefringent properties of several LCs (5CB, E7) operating from microwave to visible range. All dielectric properties were characterized at room temperature.

	Frequency range	*n*_e_	*n*_o_	Δ*n*	tan*δ*_e_	tan*δ*_o_	ss	Ref.
5CB	Microwave (19 GHz)	1.732	1.643	0.009	0.0132	0.0273	aa	[200]
	Terahertz (1.06 THz)	2.043	1.807	0.236	0.045	0.066	aa	[201]
	Infrared (4.45 μm)	1.652	1.499	0.153	0.1961	0.0634	aa	[202]
	Visible (633 nm)	1.719	1.528	0.191	–	–	aa	[203]
E7	Microwave (30 GHz)	1.791	1.652	0.0139	0.0317	0.0486	aa	[204]
	Terahertz (1.0 THz)	1.704	1.562	0.142	0.0139	0.0429	aa	[202]
	Infrared (10.6 μm)	1.69	1.49	0.2	0.004	0.0004	aa	[205]
	Visible (633 nm)	1.731	1.519	0.211	–	–	aa	[206]

The modulation speed of liquid crystals is also an important factor for the evaluation of dynamic switching of modulators. The modulation speed of LCs is determined by the switch-on time constant *τ*_on_ and the relaxation time constant *τ*_off_ [197]. Both time constants depend on the cell thickness *d* with *τ* ∝ *d* ^2^. However, the switch-on time constant is strongly related to the applied voltage, i.e. $\tau \propto 1/\left(V^{2}-V_{c}^{2}\right)$ , while the relaxation time constant only depends on the property of the LC, including the nematic viscosity *γ* and elastic constant *K* [207]. Therefore, direct approaches to increase the modulation speed include using a thinner LC cell, a larger external bias, selecting LCs with smaller visco-elastic ratio *γ*/*K*, and operating at an elevated temperature. However, while a higher temperature can be used to decrease the visco-elastic ratio, it can lead to smaller birefringence and larger loss [208]. A recent demonstration has also shown that it is possible to decrease the visco-elastic ratio by filling 5CB into a patterned polyimide cell which provides an increase of the anchor area for the 5CB mesogens. As a result, the modulation frequency to obtain largest frequency shift is ∼1 kHz as shown in Figure 7(C) and D [90]. An increase of the modulation frequency of twisted nematic LCs was demonstrated to about 2 kHz by doping metal particles [209].

Infiltrating liquid crystal into the metamaterials enables customization the electromagnetic response through changing the local environment of resonators. The first experimental implementation was achieved in the X band to tune the permeability of the SRRs [91]. Since then, liquid crystals have been used to reconfigure various properties of metallic metasurfaces, including negative refractive index [210], absorption [90, 211], phase [212, 213], and polarization conversion [92, 214]. LCs can also be used to modify the mode properties of all dielectric metasurfaces, which depend on the index contrast between dielectric material and the surrounding matrix. As shown in Figure 7(E) and (F), by loading a thermally tunable liquid crystal onto a near-infrared all-dielectric metasurface, a temperature increase from 20 °C to 60 °C resulted in a maximum resonance shift to shorter wavelengths with 40 nm and a significant change on the transmission by a factor of five [199]. Electrical tuning can also be implemented in the visible and near-infrared range using ITO as electrodes [215, 216]. The ease of fabrication and integration and scalability of liquid crystal based metasurfaces have enabled practical applications and devices. This includes projection [217], beam steering [211, 218, 219], and devices such as a spatial light modulator [220, 221] and tunable metalens [222].

#### 3.2.5 Micro-electro-mechanical systems

Micro-electro-mechanical systems (MEMS) are a technology using micro-/nano-fabrication techniques to create miniature devices combining mechanical and electrical components on a single chip. MEMS actuators, which provide mechanical change using physical forces, have been widely used for optical modulation. There are myriad MEMS devices but bimaterial cantilevers/beams, electrostatic-driven actuators, and piezoelectric actuators are the most commonly used active components in MEMS actuators.

**Figure 8:**
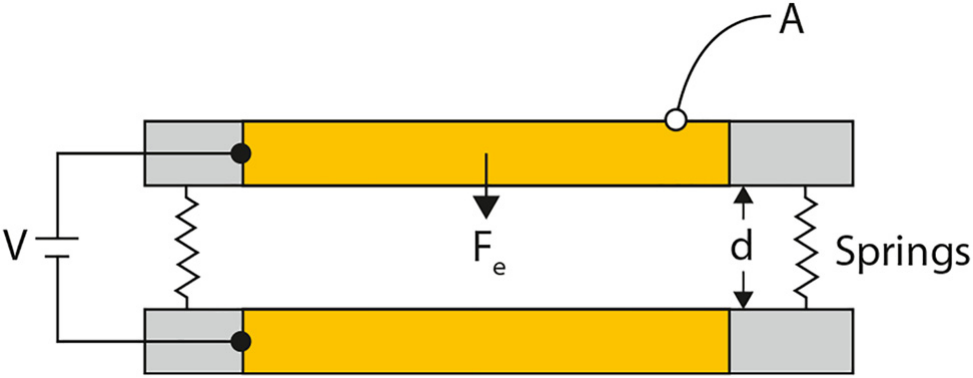
Schematic of electrostatic attraction through application of a voltage (V) across two conductors (gold), shown in cross-section. The top conductor (gold) is mounted to a nonconductive platform (gray), both of which are movable, where the springs provide a restoring force. The conductors have area (A) with an equilibrium separation distance (d).

Cantilevers and beams are the simplest mechanical elements used for actuation. Cantilevers can be driven in different ways that depend on the materials and geometry. This includes thermal bending using bimaterials [223], electrostatic driving using conductive materials [224], and vibration using piezoelectric materials [225]. In MEMS or nanoelectromechanical system (NEMS) devices, one actuation mechanism is that of electrostatic attraction (EA) or repulsion due to electrostatic forces between charged structures [226]. In Figure 8 we show a schematic cross-section of an MEMS device where EA is used to move a conducting segment (top gold) with regard to a fixed (stationary) conductor (bottom gold). When a voltage **V** is applied, electrostatic force **F**_e_ results, which moves the top conductor via the Lorentz force, given as,

(8) $$F_{e}=QE$$

where the electric field is *E* = *V*/*d*, *Q* is the charge, and *d* is the distance between the two conductors. If the conducting regions of the parallel plate MEMS shown in Figure 8 each have an area *A*, and form the MEMS capacitive portion, then the capacitance may be modified through application of a voltage through,

(9) $$C\left(V\right)=\epsilon_{0}\frac{A}{d\left(V\right)}$$

where the distance *d* = *d*(*V*) is a function of applied bias due to electrostatic attraction.

**Figure 9:**
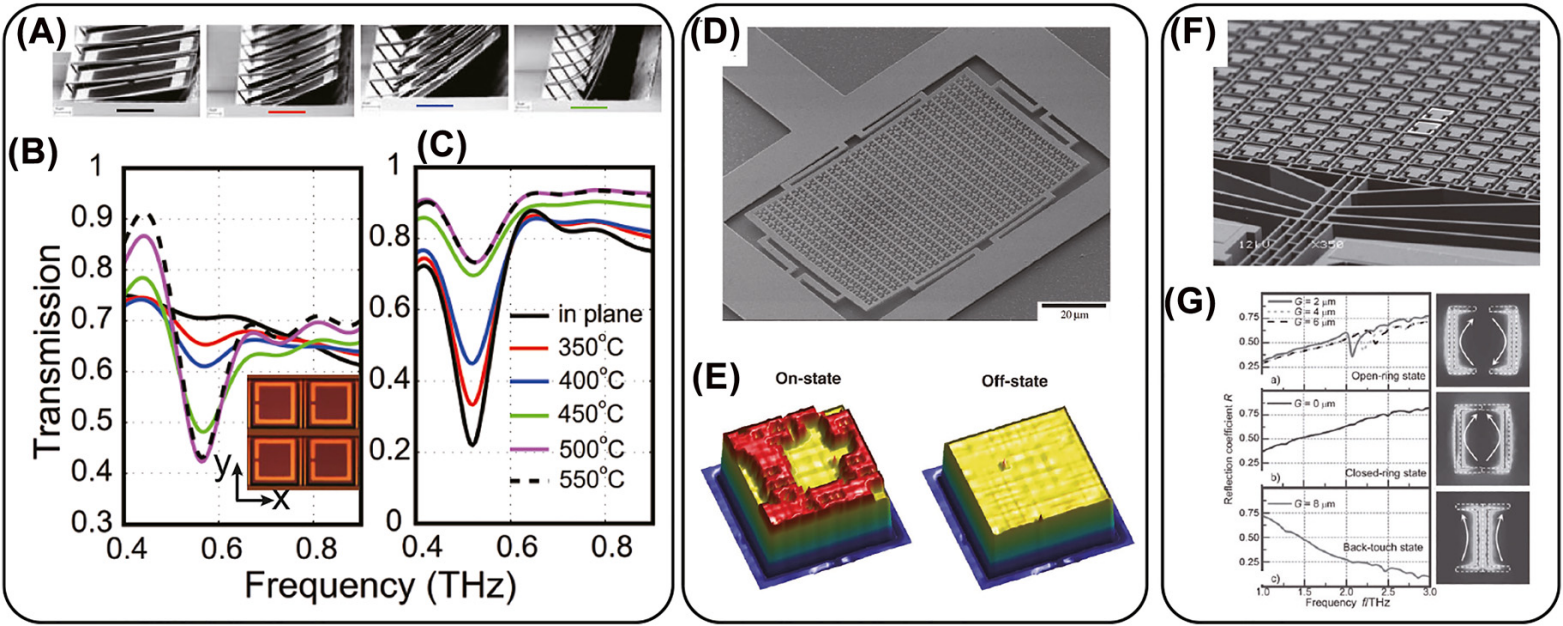
MEMS based tunable metasurfaces. (A)–(C) Reconfigurable terahertz metamaterials. (A) SEM images of metamaterial array rotated with the bimaterial beams annealed at various temperatures. THz transmission response of the SRR changing with the rotation angle for magnetic field along the *y*-direction (B) and electric field polarized along the *y*-direction (C). The inset in (B) is the microscope image of the as-fabricated SRRs before annealing process. (A)–(C) Reprinted from [93]. Copyright 2009, American Physical Society. (D) SEM image of an MEMS metasurface emitter. (E) Measured emitted power density of an 8 × 8 dynamic emitter array with an IR camera in on and off states. (D) and (E) reprinted from [232]. Copyright 2017 Optica. (F) SEM image of a fabricated comb-drive actuated magnetic metamaterial. (G) The measured reflection coefficient of the metamaterial with different open-ring states from gap size *G* = 0 μm to *G* = 8 μm. *G* = 0 μm and *G* = 8 μm are closed-ring state and back-touch state, respectively. (F) and (G) reprinted from [233]. Copyright 2011 John Wiley & Sons.

Thermally actuated bimaterial cantilevers or beams can provide large deformation and continuous modulation. Because of the difference between the thermal expansion coefficients, a temperature change leads to a significant stress difference in the two layers, and subsequent beam deformation. Based on a mechanical analysis, the out-of-plane displacement for a free-end bimaterial cantilever is expressed as [227]

(10) $$\Delta z=\frac{3\Delta \alpha \Delta TL^{2}}{t_{\text{top}}}\frac{\eta \xi^{2}\left(\xi +1\right)}{\left(1+\eta \xi \right)\left(1+\eta \xi^{3}\right)+3\eta \xi {\left(1+\xi \right)}^{2}}$$

where *δα* = *α*_top_ − *α*_bottom_ is the difference of thermal expansion coefficient between the top and bottom thin films, Δ*T* is the temperature change from the initial state, *L* is the length of the cantilever, *η* is the ratio of the Young’s Modulus of the top and bottom layers, *ξ* is the ratio of the thickness between the top and bottom layers. By selecting materials with drastically different thermal expansion coefficients, the displacement of the cantilevers can be as large as hundreds of micrometers [228]. The direction of the bending can also be controlled via the sequence of stacking thin films with positive or negative values of Δ*α*. An initial attempt to modify the coupling between the metasurface and the incident wave was achieved through a rapid annealing process on bimaterial actuators with gold coated on silicon nitride beams, as shown in Figure 9(A)–(C) [93]. By increasing the annealing temperature to about 550 K, the tilting angle of the split-ring resonators is nearly vertical to the horizontal plane. This reorientation is such that the resonators couple to the magnetic field component of the incident THz radiation in contrast to when the resonators are in-plane. However, such a thermal process is not reversible because of the permanent stress change in the bimaterials. Therefore, approaches using direct heating via thermal and electrical techniques have been implemented to achieve dynamic tuning on the metamaterials [229–231]. Electrothermal actuation advantages for dynamic control and compatibility with CMOS technology. However, joule heating requires considerable power and the response of the thermal bending is relatively slow. For a bimaterial device operating in the terahertz range with beam length of about 415 μm, the modulation frequency is about 25 Hz [122].

Electrostatic driving is one of the most attractive methods for MEMS devices because of the compact device, repeatable mechanical deformation, lower power consumption, and easy interfacing with CMOS control circuits. The electrostatic force is dependent on the distance of the two plates, and is balanced by the restoring force from the bending or rotation of beams. As the applied voltage passes a critical point, i.e. pull-in voltage, the two plates will collapse together. And the traveling distance for the moving plate is only 1/3 of the initial gap size of the two plates. Because of the limited displacement range, electrostatic force induced bimaterial bending is mainly used for modulating devices operating in the range from terahertz to visible with out-of-plane deformation, including resonance switching [94, 234], [235], [236], [237], amplitude modulation [238–241], phase modulation [242, 243], electromagnetically induced transparency tuning [244, 245], chirality control [246, 247], absorption/emission control [95, 232], and phase conversion [248]. The variation of vertical distance by electrostatic actuation can also be used to tune the coupling between top and bottom photonic structures. Metal based metasurface perfect absorbers usually include a spacer layer between top metasurface layer and bottom ground plane. Changing the thickness of the spacer layer alters the induced magnetic response, which subsequently changes the absorption and resonant frequencies [232, 238]. Equivalently, the modulation of the absorptivity is the same as that on the emissivity. Figure 9(D) shows an SEM image of a dynamic infrared absorber with Babinet’s metasurfaces supported by eight beams over a gold coated silicon substrate. Applying a 15 V between the top metasurface layer and bottom ground plane, the absorptivity near the resonance of 8.9 μm increases remarkably, leading to 70% of differential absorptivity at the peak. The resulting emissivity switching is equivalent to a temperature change from 23 °C to 42 °C. In addition, such a mechanical structure provides a modulation bandwidth as large as 110 kHz, which is much faster than approaches based on direct thermal heating [249]. Through tessellating these pixels into an 8 × 8 array, programmable voltages on the pixels enable switching of the emissivity patterns as shown in Figure 9(E), which highlights the potential for applications such as thermal identification.

Since most photonic structures are fashioned in a planar configuration, the lateral displacement of the structures can be used to change the gap size, or overlap area, such that the capacitance, field enhancement and coupling between resonators can be dynamically modulated. Comb-drive actuation is most common approach capable of achieving large in-plane movement using electrostatic forces. The first demonstration of comb-drive based tunable metamaterial was achieved on a double-split ring resonators. In the arrayed structure as shown in Figure 9(F) and G, half of the ring was docked on a silicon substrate, while the other half translated with the comb-drive actuator [233]. Then, the resonant response blue-shifted as the two rings moved from gap-closed state (gap = 0 μm) to the back-touch state (gap = 8 μm). When the rings came into contact forming a single structure, the resonance disappeared as shown in Figure 9(G). Such a method can be extended to achieve anisotropic modulation through breaking the symmetry of a maltase cross metamaterial thereby achieving polarization control in the terahertz range [250]. In addition, the in-plane translation of photonic structures can also be used to modify the nearest-neighbour coupling between resonant structures, achieving group delay modulation [251–253].

Piezoelectric actuation is another approach widely used in MEMS technology to achieve micro-/nano-scale mechanical driving. Piezoelectric materials, such as barium titanate (BTO), lead zirconate titanate (PZT), AlN, ZnO, polyvinylidene fluoride (PVDF), allow electric potential generation in response of applied mechanical stress, and vice versa. A straightforward approach is to directly pattern arrayed resonators and a metallic ground plane on alternate sides of a piezoelectric slab. With an applied bias, the piezoelectric effect produces an out-of-plane deformation on the slab, thereby leading to resonant frequency tuning and a phase shift [254, 255]. Conversely, the piezoelectric effect can also be used to modify the resonant mechanical frequency for electromagnetic wave detection using a plasmo-mechanical coupled piezo-metasurface integrated structure [256].

In addition to the major reconfiguration approaches mentioned above, there have been other MEMS-based modulation methods for obtaining reconfigurable EM responses, such as mechanical actuation using electroactive *π*-conjugated polymer polypyrrole (PPY) [257], pneumatic modulation through creating pressure difference across a device [258], mechanical deformation based on shape memory alloy (SMA) [259], and reconfiguration based on a microfluidics metasurfaces [260]. For further details, the readers can refer to specific review work on MEMS integrated metamaterials [261–263].

#### 3.2.6 Other active metamaterials

**Figure 10:**
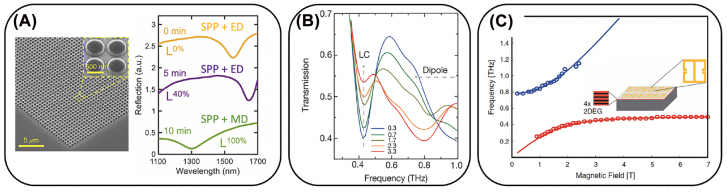
(A) Tilted SEM image of combined Si nanodisk Au plasmonic structure with integrated GST phase change material (the cross-section is shown above in Figure 3B, lower left). The right part of the figure shows experimental data with thermal annealing at 145 °C for 0, 5, and 10, minutes, corresponding to a change from amorphous GST to crystalline GST. With increasing crystallinity and the corresponding change in the GST refractive index the surface plasmon – dipole coupling changes from electric dipole (ED) to magnetic dipole (MD) with an initial redshift at 5 min and subsequent blueshift 10 min. Reprinted from [78] Copyright 2021, ACS (B) Nonlinear transmission as a function of frequency for split ring resonators (SRRs) on VO_2_ for various in-gap peak terahertz electric fields as denoted in the inset (in MV/cm). This data is for the structure shown in Figure 2D. With increasing field strength, the transmission dip at ∼0.43 THz is reduced in magnitude, arising from field-induced tunneling and subsequent Joule heating which drives the insulator-to-metal transition on a ps timescale, shorting the capacitive regions of the SRRs. All of the data is in this figure is taken below the damage threshold (∼4 MV/cm), in contrast to Figure 2D, where the damage threshold was purposely exceeded to highlight the localized field enhancement. Reprinted from [54], Copyright 2012, Nature Publishing Group. (C) Strong coupling of the cyclotron resonance of a GaAs two-dimensional electron gas (2DEG) with the dipole resonance of an SRR. A large splitting of the lower polariton (data - red dots) and upper polariton (data - blue dots) as determined from terahertz transmission measurements as a function of magnetic field which sweeps the cyclotron resonance through the bare SRR resonance. The lines are model fits, and the inset shows that for this particular data a four layer 2DEG is utilized to increase the coupling. Reprinted from [84], Copyright 2012, AAAS.

Beyond what has been presented in the previous sections, there are exciting demonstrations and opportunities with numerous other active metamaterials to both investigate fundamental light–matter interactions, and to realize novel metamaterial devices ranging from reconfigurable reflectors to novel quantum emitters. We briefly mention these topics, with some representative examples shown in Figure 10.

Phase change materials offer opportunities across the spectrum with, as mentioned in Section 3.1, GST and VO_2_ being of particular interest given the potential for room-temperature operation, large changes in the dielectric permittivity with the temperature that can be controlled with various stimuli, and integrability with metamaterial structures arising from the ability to grow high-quality films [106, 114, 264]. Figure 10(A) shows an example where GST is used to control the coupling between surface plasmons and dipolar modes of Si nano-resonators (the cross-section of this device was presented in Figure 3(B) in the lower left) [78]. Thermal annealing modifies the GST from amorphous to crystalline, yielding a change in the permittivity that, in turn, modifies the surface plasmon interaction from the electric dipole to the magnetic dipole of the Si resonators, resulting in a redshift of the absorption. Active control of the reflection from specular to first-order diffraction was also demonstrated using a multi-resonator supercell [78]. GST has also been utilized to create flexible metamaterial structures with a tunable Fano-response at terahertz frequencies [265]. Additional coverage of phase change metamaterials can be found in the complementary review by Xiao, et al. [266]

The potential for nonlinear metamaterials was realized at the outset and there were early important explorations of the possibilities describing bistability, parametric amplification, nonlinear permeability, nonlinear pulse propagation and others [52, 107, 267, 268]. Recent demonstrations include nonlinear polarization control, beam steering, and holography [111, 269], [270], [271]. We note that active nonlinear metamaterials include resonant and nonresonant phenomena, which include nonlinear carrier generation in semiconductors and phase change materials. Figure 10(B) shows an example of THz electric field switching in a split ring resonator array fabricated on a VO_2_ film which exhibits an insulator-to-metal (IMT) transition at 335 K (Figure 2(D) shows a portion of the device) [54]. With increasing field, the capacitive gaps are shorted out as the IMT is initiated through field induced tunneling and Joule heating on a picosecond timescale. This can be considered as a nonlinear response of a phase-change material. The transition occurs at fairly large in-gap fields (∼1 MV/cm). However, with judicious design of quantum materials, it should be possible to realize (through doping, strain, etc.) materials that are at the edge of instability, dramatically reducing the field required to achieve a functional nonlinear response.

The restriction of an electron gas from a third spatial direction enables the formation of a two-dimensional electron gas (2DEG), which has been shown useful for imbuing nanophotonic metamaterials with dynamic properties. We have already discussed graphene in Section 3.2.2, which is inherently a thin solid material, and therefore naturally confines electrons to two-dimensions. 2DEGs may be formed in various material systems, and high-electron-mobility transistors (HEMTs) are a common example. GaAs supports 2DEGs as metal-oxide-semiconductor field-effect transistors (MOSFETs) [272]. Perhaps more surprisingly, a 2DEG may be formed at the interface of two wide-gap insulating materials – for example in LaAlO_3_/SrTiO_3_ [273], and Mg_*x*_Zn_1−*x*_O/ZnO [274], being two examples [275]. HEMTs have been integrated into nanophotonic metamaterials to provide active control with demonstrations of 10 MHz frequency modulation (*f*_mod_) of THz waves [79], and subsequent demonstration of THz modulation at *f*_mod_ = 1 GHz [276], and *f*_mod_ = 3 GHz [277]. For a good review of 2DEGs in electromagnetic metamaterials see [278] and references therein.

Dynamic metasurfaces using chemical stimuli have been demonstrated. A common material of choice is magnesium (Mg) [279], which undergoes a phase transition through hydrogen uptake to magnesium hydride (MgH_2_), which is reversible upon oxygen exposure. The dynamic properties of Mg-based nanophotonic metasurfaces arise because Mg is a metal and MgH_2_ an insulator [280]. Therefore through cycling of hydrogen and oxygen, a switching between conductive and insulating states can be achieved. A similar approach based on electrochromism uses electrical stimuli to induce electrons and ions (H+, Li+) to enter into redox-active host material such as tungsten trioxide WO_3_. The net effect is a drastic change in the conductivity by several orders of magnitude [281]. Chemical stimuli have enabled dynamic properties to be achieved in nanophotonic plasmonic metasurfaces demonstrating the ability to achieve chiral polarized light and singular beams in the visible [282, 283], and color displays [284–286].

As a final example, we mention the role of metamaterials for strong-coupling studies of quantum materials. As mentioned in Section 3.1, a resonant material response such as a cyclotron resonance in a two-dimensional electron gas (2DEG) can be tuned (with a magnetic field in the present case) to coincide with a metamaterial resonance [84, 85, 287]. This can lead to polaritonic physics where, for sufficiently strong coupling, energy exchange between the material resonance and the cavity results in hybridization creating modes of mixed character [82, 86]. Cavity polariton has long been investigated at visible frequencies using high-quality semiconductor heterostructures [288, 289]. With developments in metamaterials, this has led to new possibilities including accessing the ultrastrong coupling regime where the coupling energy exceeds the bare cavity resonance energy [86]. This leads to exotic possibilities including a ground state composed of virtual correlated photon pairs that, upon rapid quenching of the coupling, would lead to novel photon emission [85]. A canonical example is presented in Figure 10(A) where marked splitting arises from the coupling of a four-layer 2DEG to the SRR resonance as determined from terahertz transmission measurements as a function of magnetic field [84]. The coupling is approximately 60% of the bare cavity strength in this case, and optimization has achieved greater-than-unity coupling [85]. These structures are essentially novel active materials achieved through strategic integration of high-quality quantum materials with split ring resonators. Finally we note the possibility of using metamaterials to tune the properties of other quantum materials such as superconductors. For example, metamaterials could suppress fluctuations of the superconducting order parameter leading to an increase in the transition temperature [287, 290, 291].

## 4 Devices and applications

In this section we highlight examples of active and tunable nanophotonic metamaterial devices and their applications. In Section 2 we detailed features of electromagnetic materials that make them a novel and ideal platform for photonics – that is MMs are resonant effective media, multifunctional, subwavelength, and additionally follow electromagnetic similitude. Many of the devices and applications discussed here take advantage of these metamaterial features and the intuitive design paradigm afforded by metamaterials.

**Modulation applications:** The modulation of electromagnetic waves is important in many photonics devices and applications. For example, much of the technology we rely on today is based on digital communications, and thus would not be possible without carrier frequency modulation [292]. Modulators were one of the first examples demonstrating the dynamic capabilities of metamaterials [100, 293], [294], [295], and there have since been numerous examples operating across the spectrum [296, 297]. More generally, metamaterials have demonstrated modulation of transmission [294], reflection [298], absorption [220], polarization [299], and surface electromagnetic waves [300]. Metamaterial modulators have been used for terahertz time-domain spectroscopy [301], formed into spatial light modulators (SLMs) [302] for single pixel imaging [303], near-field imaging [304], arranged linearly [305] or in an array [306] as elements for electronically steered synthetic aperture radar (SAR). Metamaterial modulators have found many other uses including equivalent time sampling [155], and computational spectroscopy [307].

The external stimuli utilized for metamaterial modulation include electronic [88], optical [100], thermal [308], and non-linear properties [107], in various materials systems such as semiconductors, two-dimensional materials, and oxides. MEMS may be used as elements located in strategic locations to enable modulation [293], or the entire metamaterial array may be moved to achieve modulation [123, 252]. As discussed, ultrafast optical pulses were used to show modulation of transmission of metamaterials formed on a GaAs substrate at THz frequencies [100], while n-doped GaAs permitted demonstration of all-electronic modulation the same year [88].

**Figure 11:**
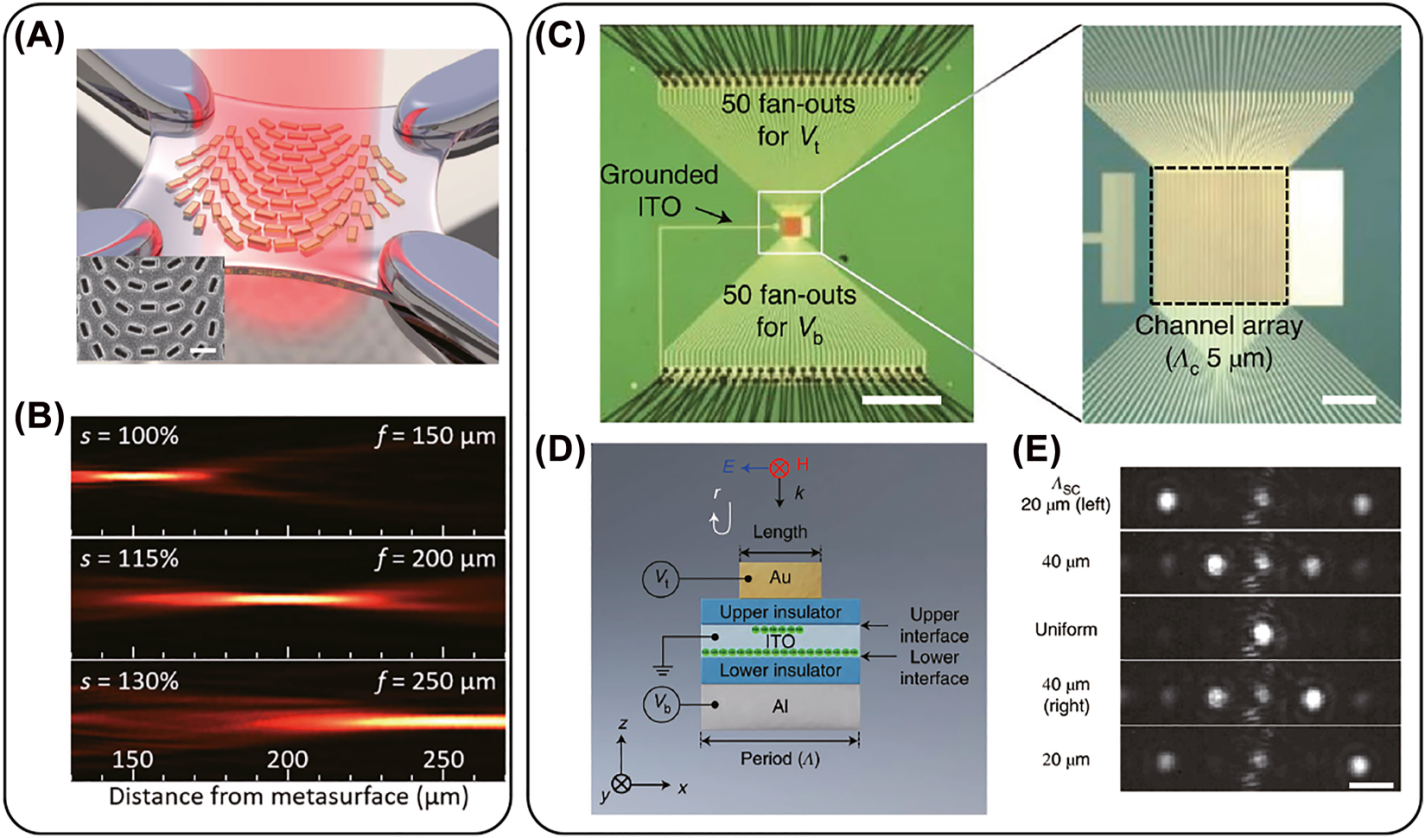
(A) Illustration of a stretched visible meta-lens. The inset shows the fabricated plasmonic gold nanorods. (B) Experimental results of a 1.7× zoom lens with strain tuned by 30%. (A) and (B) reprinted from [121]. Copyright 2016 American Chemical Society. (C) Microscopic image of an active array with 50 top and 50 bottom terminals. Scale bar, 1 mm. The right image shows a magnified view the active device located in the middle of the fan-outs with a size of 250 × 250 μm^2^. Scale bar, 100 μm. (D) Schematic view of the cross section of a single plasmonic nanoresonator, which consists of top gold nanoantenna, insulator spacer and the active ITO layer in the middle. The bottom layer is an Al mirror. (E) Measured intensity profiles for five different steering angles showing linear movements of the focus beam. (C)–(E) reprinted from [315]. Copyright 2020, Springer Nature.

**Dynamic Lenses:** A meta-lens is a flat optical device, which is able to manipulate the phase, amplitude and polarization of light through wave front control at the subwavelength scale. With optimization of the meta-atom configuration, meta-lenses have been demonstrated to achieve diffraction-limited focusing, chromatic aberration correction, and high-focusing efficiency with a large numerical aperture [309–312]. Dynamic tuning of these properties could enable novel and more compact optical systems exhibiting multifunctionality. With an assumption of similar scattering efficiency for each meta-atom, a typical flat meta-lens can be generally constructed by following the phase profile of a Fresnel lens as,

(11) $$\varphi \left(\rho ,\lambda \right)=\frac{2\pi }{\lambda }\left(\sqrt{\rho^{2}+f^{2}}-f\right)$$

where *ρ* is the distance to the center of the lens, *λ* is the operating wavelength, and *f* is the focal length. To achieve dynamic tuning of the focal length, several strategies have been demonstrated to modify the phase profile, including dynamic scaling of the lens using elastic polymers, local modulation of each meta-atoms, tuning on a combined meta-lens set. A paraxial phase profile of a lens can be approximated as *φ*(*ρ*, *λ*) ≈ *πρ* ^2^/*λf*. An isotropic scale of the phase profile, such as uniformly elongating metasurfaces by a stretching ratio of *η*, would equivalently result in an increase of the focal length by (1 + *η*)^2^ [313]. The initial demonstration of tunable meta-lens was obtained by arranging rotated gold nanorods on an elastic PDMS substrate as shown in Figure 11(A) [121]. Due to the large strain limit of PDMS, the focal length can be continuously tuned from 150 μm to 250 μm as the relative positions of the plasmonic gold nanorods change with the elastic deformation of the substrate. Beyond plasmon-based verifocal meta-lenses, high-performance adaptive meta-lenses with astigmatism and image shift corrections can also be achieved through patterning dielectric meta-atoms on a dielectric elastomer actuator (DEA) [314]. Instead of directly using mechanical forces to stretch the substrate, the DEA works as a compliant parallel plate capacitor, which stretches under a large electric field. With an applied voltage as high as 3 kV, 100% focal length tuning is realized.

In addition to strain engineering, various external stimuli, such as MEMS, liquid crystals, and thermal control on phase change materials were also used to realize varifocal meta-lenses. MEMS structures can provide precise spatial phase control; large movement and fast modulation speed and are easier for integration. Using a comb-drive structure, the orientation of the meta-lens surface can be tilted along the two orthogonal axes by ±9*°*, thereby rotating the focal point [316]. Electrostatic MEMS systems with lateral or vertical displacement can also be utilized to achieve focal length change with tuning the gap distance or the overlapped area between two stacked lenses [317–320]. A proof-of-concept varifocal meta-lens doublet was first demonstrated through electrostatic modulation of the distance between two infrared meta-lenses. Upon 1 μm out-of-plane displacement with a modulation speed of a few kHz, over 180 diopters change in the optical power and over 60 μm variation of the focal length can be obtained [317]. According to Eq. (11), as long as the phase delay at each position changes, the focal point will correspondingly shift. Thus, a substrate index change would also lead to a varifocal meta-lens. PDMS and liquid crystal have both been employed to obtain tunable meta-lenses through changing their refractive index [222, 321]. Further, because of large birefringence, liquid crystals can also be used for polarization dependent phase control over the metasurface, which enables a new freedom for meta-lens control, such as polarization multiplexing for three-dimensional switching of the focal spots [322, 323]. Varifocal meta-lenses based on a refractive index change can also be carried out by incorporating phase change materials [324, 325]. However, different from other techniques with continuous change of focal length, meta-lens based on PCMs normally lead to a focal length switching due to the material phase switching between amorphous and crystal states, i.e. a bifocal meta-lens.

Since the meta-lens requires spatially varying phase and constant reflectance or transmittance, each meta-atom has to be optimized with a specific phase and amplitude. When the parameter space is small, a conventional parameter sweep can be performed for target designs. However, for a complex design with multiple parameters, it is computationally expensive using an ergodic search method. Therefore, novel design strategies, such as a genetic design approach, or a [325] adjoint gradient method [326], have been proposed to mediate the intensive simulations without degradation in performance. Furthermore, the emergence of deep learning techniques is also promising to inversely design desired dynamic meta-lenses.

**Reflect- and transmit- Arrays (RTAs):** Similar to dynamic meta-lenses, RTAs are also active devices used for wave-front control by dynamically directing electromagnetic waves into target directions while keeping the reflection or transmission as high as possible. Reflectarrays were initially prevalent in the microwave range for radar applications. In recent years, with the progress on the photonics and metasurfaces, reflectarray techniques have been extended from terahertz to optical frequencies due to their potential applications for wireless communication, self-driving with LiDAR, imaging, and holography. An ideal RTA can be considered as a dynamic gradient metasurface whose phases are spatially distributed with gradient in a supercell. Therefore, the diffraction angle of the scattered wave relative to the incident wave can be described as

(12) $$n_{t}\sin\theta_{r,t}-n_{i}\sin\theta_{i}=\frac{\lambda }{2\pi }\frac{\text{d}\Phi }{dx}$$

where *θ*_*i*_ is the incident angle, the subscripts of *θ*_*r*,*t*_ represent the reflected or transmitted angle, respectively, *λ* is the working wavelength and dΦ/d*x* indicates the gradient of the phase discontinuity at the interface. Thus, steering the diffracted beam can be realized through engineering the phase gradient. A metamaterial reflectarray was initially demonstrated as a metamaterial leaky-wave aperture with frequency-dependent radiation patterns for compressive imaging [16, 327]. As active elements are hybridized with the subwavelength meta-atoms, the “on” and “off” response due to external stimuli on those active elements could give rise to over 180-degree phase change [185, 328], [329], [330], [331]. Up to the present, various excitation methods have been applied to realize different active elements for beam steering, such as electrical tuning using semiconductors, graphene, and ITO thin films [191, 332], [333], [334], electrical or thermal trigger on liquid crystal and PCMs [211, 219, 221, 335], [336], [337], electrical or mechanical tuning using MEMS structures [318, 320, 338].

For most dynamic metasurfaces, the modulation was only achieved by a single external control. Such a single modulation scheme normally brings about a change of amplitude and phase simultaneously. This correlated modulation could deteriorate the beam deflection efficiency because of the spatially variation of the amplitude in a reflective array [185]. Therefore, a dual-gated control approach was proposed to independently modulate the phase and amplitude of the reflection [315, 330]. Figure 11(C) shows an optical image of an electrically tunable dual-gated reflective array with 50 top and bottom control terminals, respectively. Such a reflective array consists of 550 individual addressable nanoresonators with the unit cell shown in Figure 11(D). Tuning was achieved by applying separate electrical biases to the top and bottom electrodes to change the carrier density at the two ITO/dielectric interfaces. Because of the independent control on amplitude and phase, a complete phase modulation close to 360*°* was obtained while the reflection amplitude remains around 4%. Figure 11(D) shows the measured beam steering to different diffracted angles. As an alternative approach to increase the beam steering efficiency, the correlated variation of amplitude and phase has been taken into account simultaneously using phased array equations [339]. In recent years, state-of-the-art algorithms for a supercell-level design, such as genetic algorithm, adjoint inverse design, and deep learning, have been explored to tackle this problem [340–342]. In addition to the applications mentioned above, dynamic metasurfaces have demonstrated capability for many other novel applications, including gas sensing, information encryption, and optical communications [343–345].

## 5 Summary

During the past decade, metamaterials have emerged as state-of-the-art photonic and nanophotonic materials. The intuitive design paradigm that underpins metamaterial research has enabled the demonstration of multifunctional exotic electromagnetic properties which span the electromagnetic spectrum. The ability to scale resonant metamaterial responses to nearly any band of the spectrum has facilitated the demonstration of exotic properties at short wavelengths that have often been initially verified at longer wavelengths. The accuracy of computational electromagnetic simulations and maturity of nanofabrication has aided state-of-the-art demonstrations of metamaterials as photonic materials and devices with impressive technological potential. The ability of metamaterials, through numerous integration strategies, to access exotic electromagnetic responses difficult to achieve with naturally occurring materials bolsters their prospects for novel photonic applications.

While static metamaterials have led to impressive photonics advances, the metamaterials design paradigm also provides a powerful approach for the realization of functional composites capable of controlling the amplitude, phase, polarization, wavelength, and directionality of electromagnetic waves while enabling cross-fertilization with novel materials and systems offering the promise of continued growth and significant future opportunities. As the examples in this review demonstrate, the multifunctional capability of metamaterials permits electromagnetic properties to be modified in real-time, providing an enormous level of functionality to help solve outstanding grand challenges in nanophotonics.

Dynamic 2D metamaterials (i.e. metasurfaces) which include frequency agile and spatiotemporal metasurfaces and gradient index metasurfaces were demonstrated over fifteen years ago [20]. Active photonic metamaterials have certainly advanced far beyond these initial discoveries, but there is still much to explore. Indeed, there are many nanophotonics technologies for which dynamic capabilities will play a key role, and we close by highlighting a few. Next generation wireless systems will rely greatly on active metasurfaces, and therefore further developments in the intelligent dynamic control of polarization, frequency, and directionality will be of paramount importance. For example, the creation of adaptive and reconfigurable surfaces, termed reconfigurable intelligent surfaces (RIS) [346–350], or intelligent reflective surfaces or “smart radio” is an exciting and fast growing “active nanophotonics” area. Future dynamic metasurfaces will enable sculpting of the electromagnetic environment through directional reflection, absorption, and channel combining for next generation wireless systems. Faster wireless will enable streaming from the cloud where people can use extended reality (XR) devices – encompassing virtual reality, augmented reality, and mixed reality – regardless of their computational capability, location, or time zone. The XR devices themselves will benefit from real-time tunable nanophotonic metalenses with holographic displays [30, 351], [352], [353], [354].

The field of structured light deals with singularities in the phase or polarization of light beams and may have applications toward diffraction-less or “self-healing” propagation, particle acceleration, and single molecule spectroscopy [355, 356]. Metasurfaces have been used to realize structured light and enable the construction of compact sources of beams with exotic angular momentum states – consisting of spin angular momentum (SAM) and orbital angular momentum (OAM) – permitting applications including imaging [357], communications [358], and quantum encryption [359]. Indeed, structured light phenomena and the associated potential applications will likely benefit from future advances in nanophotonic metasurfaces with dynamic capabilities.
